# Fin Whale Sound Reception Mechanisms: Skull Vibration Enables Low-Frequency Hearing

**DOI:** 10.1371/journal.pone.0116222

**Published:** 2015-01-29

**Authors:** Ted W. Cranford, Petr Krysl

**Affiliations:** 1 San Diego State University, Department of Biology, San Diego, California, United States of America, and Quantitative Morphology Consulting, Inc., San Diego, California, United States of America; 2 University of California San Diego, Department of Structural Engineering, La Jolla, California, United States of America; Institut Pluridisciplinaire Hubert Curien, FRANCE

## Abstract

Hearing mechanisms in baleen whales (Mysticeti) are essentially unknown but their vocalization frequencies overlap with anthropogenic sound sources. Synthetic audiograms were generated for a fin whale by applying finite element modeling tools to X-ray computed tomography (CT) scans. We CT scanned the head of a small fin whale (*Balaenoptera physalus*) in a scanner designed for solid-fuel rocket motors. Our computer (finite element) modeling toolkit allowed us to visualize what occurs when sounds interact with the anatomic geometry of the whale’s head. Simulations reveal two mechanisms that excite both bony ear complexes, (1) the skull-vibration enabled bone conduction mechanism and (2) a pressure mechanism transmitted through soft tissues. Bone conduction is the predominant mechanism. The mass density of the bony ear complexes and their firmly embedded attachments to the skull are universal across the Mysticeti, suggesting that sound reception mechanisms are similar in all baleen whales. Interactions between incident sound waves and the skull cause deformations that induce motion in each bony ear complex, resulting in best hearing sensitivity for low-frequency sounds. This predominant low-frequency sensitivity has significant implications for assessing mysticete exposure levels to anthropogenic sounds. The din of man-made ocean noise has increased steadily over the past half century. Our results provide valuable data for U.S. regulatory agencies and concerned large-scale industrial users of the ocean environment. This study transforms our understanding of baleen whale hearing and provides a means to predict auditory sensitivity across a broad spectrum of sound frequencies.

## Introduction

Mysticete whales are the largest animals on Earth. The pelagic balaenopterids may reach 30 meters in length and produce low-frequency sounds in the range of 10–200 Hz [1, 2]. Most other mysticetes are primarily coastal species, less than 20 meters in length and produce sounds below 10 kHz. These acoustic frequency bands are presumably used for very long-range communication, managing social structure, and perhaps echo-navigation [3, 4].

The acoustic bandwidths used by mysticetes overlap with anthropogenic sound sources, raising concerns over potential deleterious effects from increasing trends in ocean noise [5, 6, 7, 8, 9]. Risk assessment is hampered by a lack of information about mysticete sound reception mechanisms and their sensitivity to various acoustic frequencies [10] [Science 10 January 2014, Vol. 343 no. 6167 p. 128. doi:10.1126/science.343.6167.128], [11].

To date, attempts to estimate the hearing parameters of baleen whales fall into three categories based on inferential methods: **(A)** the vocalizations of various species, based on the assumption that they can hear the sounds they generate [12, 13, 14, 15, 16]; **(B)** the anatomic structure of the ears, based on and compared to the functional morphology of the ears in well-known mammalian species [17, 18, 19, 20, 21, 22, 23]; and **(C)** behavioral reactions of wild mysticetes to playback experiments, based on the assumption that observations of behavioral reactions are interpretable in well-designed, controlled sound exposure experiments [24, 25, 26, 27, 28, 29]. Generally, these methods cannot predict mysticete sound reception mechanisms or reliably extract frequency sensitivity based on predicted sound exposure levels.

We constructed a finite element modeling system, based on serial CT scans, that allows us to predict low-frequency hearing sensitivity and identify sound reception mechanisms in cetaceans [30]. Recently, simulations from our vibroacoustic toolkit (VATk) have been validated [31, 32], used to study toothed whale bioacoustics [22, 33, 34], and hearing mechanisms in fish [35, 36]. This study shows that mysticete sound reception is primarily governed by bone conduction, as incident sound induces skull vibrations that are transmitted to both bony ear complexes.

## Materials and Methods

### Specimen

On 20 November 2003 a newborn male fin whale (*Balaenoptera physalus*) calf stranded alive on Sunset Beach in Orange County, California. Personnel from Sea World, San Diego and the California Marine Mammal Stranding Network attempted a rescue of this animal but it died during transport. This work was carried out in strict accordance with the Stranding Agreement, issued pursuant to Section 112(c) of the Marine Mammal Protection Act, between NOAA’s National Marine Fisheries Service Southwest Region (NMFS-SWR) and SeaWorld San Diego (SeaWorld) (administrative reference number 151410SWR200900478:SMW).

This stranded fin whale was 550 cm long, weighed 1,165 kg, and was assigned a Field-ID (JEH520) by John E. Heyning at the Los Angeles County Museum of Natural History. The average length at birth for Northern Pacific fin whales is between 600–650 cm, while adults can reach 2400 cm in length [37]. The necropsy was performed by Dr. Judy St. Leger with help from additional personnel at SeaWorld San Diego, the Los Angeles County Museum of Natural History, and the California Marine Mammal Stranding Network. This specimen was shorter than average for a neonate, however there was evidence of expanded lungs but no evidence of gastric milk. The umbilicus was not yet closed but fetal folds were not appreciated. This suggests an animal of a few days of age. Poor development is reasonable based on the thin nutritional condition. This suggests (but cannot confirm) to either represent placental issues or a pre-term delivery of a live calf that did not thrive due to medical concerns (Judy St. Leger, personal communication). Tissue property measurements were made by Dr. John Hildebrand and his research team from the Scripps Institution of Oceanography. The intact head was removed for further study and frozen within 24 hours of death. After sufficient time to allow for complete freezing, the head was placed inside a 48 inch diameter fiber Sonotube, and a custom container was constructed, as described previously [38]. The contained specimen was then transported to Hill Air Force Base in Utah and scanned in an industrial CT scanner. The CT data was processed into a three-dimensional image volume that provided the anatomic geometry of the animal used in our models.

After CT scanning, the head (in its container) was returned to a freezer until it was dissected on 21 August 2006. When the necropsy of the head was conducted, the tissue handling protocol was approved by the Graduate and Research Affairs, Institutional Animal Care and Use Committee at San Diego State University (APF#: 09-05-014B). Permission to possess the head was provided by a Letter of Authorization from the National Oceanic and Atmospheric Administration and the National Marine Fisheries Service Southwest Region (Administrative File: 151408SWR2013PROOOl). The prepared skull was accessioned by the Museum of Biodiversity at San Diego State University. The specimen now resides there under Accession-ID S-970.

### Computational approach

Each bony ear complex in cetaceans is a conglomeration of various bones that comprise the *tympanoperiotic complex* (TPC), which can be modeled to some approximation as a collection of vibrating solids. For example, the work reported here uses a finite element model (a complete description of the finite element model and the input parameters used in this study can be found in the S1 File). The computational problem is the so-called forced harmonic vibration analysis, which can be readily solved [39], producing results (in terms of a transfer function) that transform the incident sound pressure into some measure of input to the cochlea. The cochlear input from the stapes can be transformed into an approximation of an audiogram. In order to exercise such simulations we need to determine the forcing applied to the model of the TPC.

An incident acoustic wave of a given pressure amplitude in the sea water surrounding an animal interacts with the tissues of its head to generate traction loads on the surface of the TPC. These loadings on the TPC can be calculated from the incident sound pressure because they are, to a good approximation, driven by the amplitudes of acoustic pressure. The TPC vibrates under the action of the loads, resulting in motion of the stapes within the oval window, which produces a velocity at the center of the stapes footplate. The resulting transfer function, the Stapes Velocity Transfer Function (SVTF), is the composite of *two* transfer functions: the first transfer function calculates the pressure on the surface of TPC given the amplitude of the incident sound pressure; the second transfer function calculates the velocity at the center of the stapes footplate given the pressure on the surface of the TPC. Correspondingly, we use these two models in series to calculate the two transfer functions.

We also consider the possibility that the ossicular chain may be set into motion by loading on the TPC that is analogous to bone conduction in humans [40]. In this case, we consider that loading of each TPC can be described by the motions of the periotic bones, which are firmly embedded in the skull. Each tympanic bone is forced to follow the vibration of the periotic bone, which is set into motion by the vibration of the skull, thereby exposing the ossicles to differential displacements.

We have quantified both means of loading the TPC, by pressure delivered through soft tissues and by “skull-bone conduction”, using our two-component series of finite element models specialized for propagating elastic waves through arbitrary geometries of combined fluids and solids in an acoustic medium. The CT scans were converted to a mesh of finite elements by mapping the voxel values to the material types (see S2 Table). For the simulations reported here, incident sound waves were directed toward the head, along a single axis from directly in front of the animal at selected frequencies. At each of the excitation frequencies, after the steady-state vibration was reached, the amplitude of the total pressure in the soft tissues and the amplitudes and phase shifts of the displacement components of all tissues were extracted. These quantities were then used to define loads on the TPC as transfer functions from the sound pressure wave incident upon the animal and either the pressures acting on the TPC or the amplitudes and phase shifts of the displacements of the periotic bone.

The forced harmonic vibration analysis of the TPC then resulted in the combined transfer function between the incident acoustic pressure and the stapes footplate velocity. The stapes velocity transfer function (SVTF) was then used to estimate an audiogram, for each of the loading modalities separately and also for their combination. The audiogram curve was calibrated with respect to the minimum audible pressure using measurements and estimates from two previous studies of odontocetes [41, 42].

The sensitivity of the computed transfer functions to the input parameters was assessed by performing a series of forward/backward sensitivity analyses. The full details are provided in the S1 File.

The mechanical response of the TPC and the vibrations of the tissues of the head, especially the skull, can be visualized with animations (as shown in the S1 File).

## Results

Synthetic audiograms were generated for a fin whale head using finite element modeling simulations derived from CT scans of a small *Balaenoptera physalus*. The simulations reveal two mechanisms that excite each bony tympanoperiotic complex (TPC), the ***pressure mechanism*** and the ***bone conduction mechanism***. The ***bone conduction mechanism*** is the dominant of the two (this assertion will become clear with the figure at the end of this Results section).

The primary, or dominant, ***bone conduction mechanism*** is characterized by deformation of the whale’s skull, as the acoustic pressure waves interact with it. During ***bone conduction***, excitation of the hearing apparatus results from vibrations of the TPC induced by the motion of the skull. Secondarily, the ***pressure mechanism*** is the result of the acoustic pressure waves that reach the TPC through the seawater and various soft tissue pathways, resulting in direct pressure loading on the tympanic bulla.

**Fig 1:**
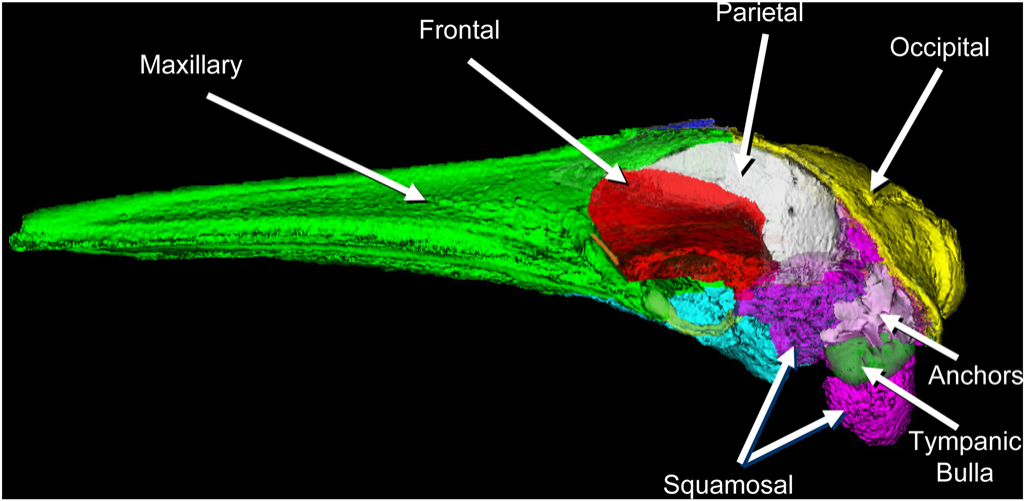
Left lateral view of the skull bones in a fin whale (*Balaenoptera physalus*). Some display transparency has been applied to the squamosal bone so that the tympanic bulla and the dense bony ossifications or “anchors” become visible. Bony skull components that are visible in this orientation are the: occipital (yellow), parietal (white), frontal (red), maxillary (green), squamosal (magenta), tympanic bullae (green), and the “anchors” (white). The dense bony anchors fan out dorsolaterally within the squamosal bones of the skull (see also Fig. 2). In this lateral view, the adjacent periotic bones are not visible because they are obscured by the anchors and tympanic bulla.

**Fig 2:**
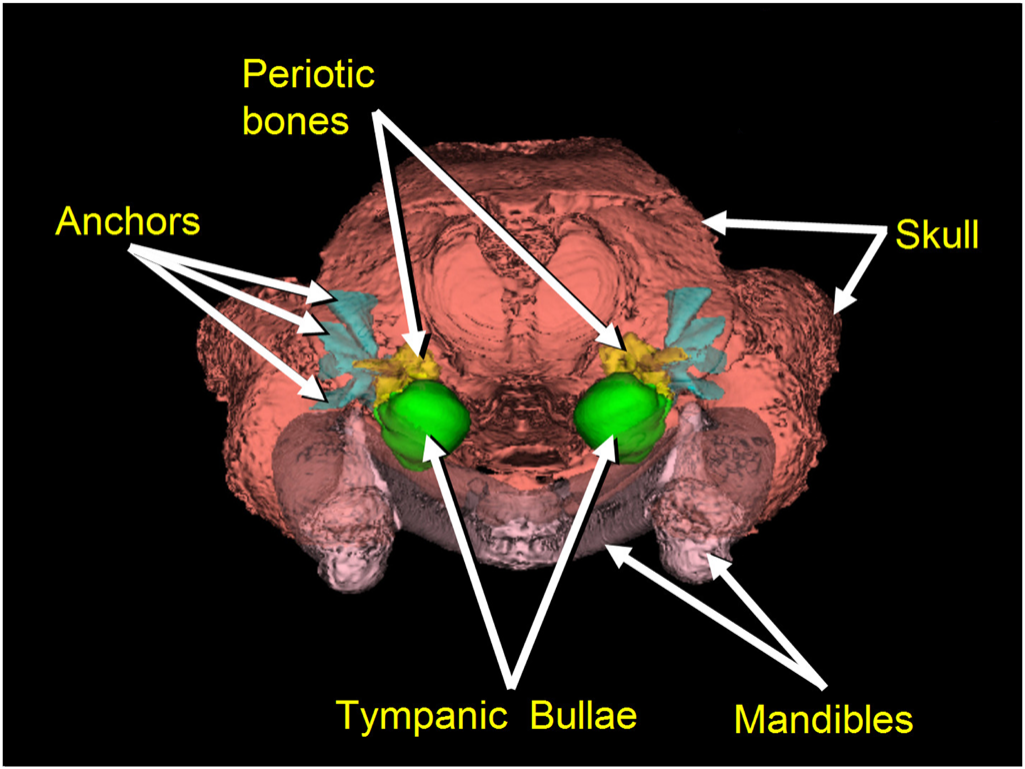
Posterior view of both tympanoperiotic complexes in a fin whale (*Balaenoptera physalus*), with some display transparency applied. The periotic bones (yellow) and tympanic bullae (green) are components of each TPC. The dense bony fan-like projections (cyan) are contained within the bones of the skull (salmon). Specifically, the anchors fan out as dense ossifications within the squamosal bone, from a locus at the junction with the juxtaposed periotic bones, and may function to stiffen the connection between the periotic and the skull. The mandibles (pink) are shown for context.

Our assertion that mysticetes receive sound by a ***bone conduction*** mechanism is buttressed somewhat by the morphology of the skull and TPC, as noted by previous authors [43, 44]. In baleen whales the posterior process of the TPC is wedged between the squamosal and the exoccipital bones, while the anterior process is sandwiched between the squamosal and the pterygoid bones of the skull [45, 46, 47, 48]. In this fin whale, the CT scans reveal that there are also a series of dense bony ossifications within the squamosal bones of the cranium that appear to fan out from the junction with the adjacent periotic portion of each TPC (Figs. 1 and 2). Upon closer inspection these dense ossification components of the squamosal bones suggest that they may function to “anchor” or extend and reinforce the connection between the TPC and the cranium (Figs. 1 and 2).

By contrast, the TPCs of most odontocetes tend to be separated from the skull. Odontocete TPCs are suspended from the skull by numerous ligamentous fibers that originate from an approximately hemispherical distribution on the periotic fossa and peribullary fossa, sometimes crisscrossing, to insert upon the periotic bone of each TPC. It is generally considered that this suspension system functions to acoustically isolate the TPCs from skull vibrations in odontocetes. Instead, sounds apparently reach odontocete TPCs primarily through the mandibular fat bodies [22, 33, 34, 49, 50, 51, 52], probably by way of the gular pathway [53]. The Physeteridae and the Ziphiidae are exceptions to this exclusively ligamentous suspension system for odontocete TPCs. The sperm whales and beaked whales retain a bony connection to the skull through the pneumatized posterior process, where the vacuities in the posterior process may be filled with lipids in life. This raises the possibility that a bone conduction mechanism may also exist in these two odontocete groups.

The results of our model have been applied to a fin whale, but the same firm connection between the TPC and the skull is common to all mysticetes [48]. This lends credence to the hypothesis that the same two sound reception mechanisms may be common to all baleen whales. However, vast differences in skull geometry between different Families of mysticetes (Balaenidae, Neobalaenidae, Eschrichtiidae, Balaenopteridae) suggest differences in the patterns of skull deformation and the resulting audiograms across these Families.

Two finite element models were required to cover the range of geometric resolution needed to accurately represent the relevant mechanisms. The first model (WP = Wave Propagation) simulated the traveling acoustic pressure waves through the water, from in front of the animal toward the head. The sound waves propagate through seawater, reach the head, continue along/within various soft tissue pathways (which have acoustic impedance values similar to water), and impinge upon the skull and the tympanic bone, the tympanic “bulla” of the TPC. The WP model could reasonably resolve the bone thicknesses of the TPC by employing cubic finite elements with dimensions of 2.7 mm on each side. This resolution was not sufficient to accurately represent the fine features of the middle ear ossicles. Therefore, the WP model was used to estimate the loading mechanisms acting on the TPC, and a second model with much higher resolution (0.684 mm) was used to compute the Forced Harmonic Vibration response of the TPC to these loads (hence the FHV model).

For the ***pressure mechanism***, forces are exerted upon the tympanic bulla by the sound pressure waves that travel through seawater and various soft tissues. The extreme impedance mismatch between soft tissue and the dense tympanic bone causes significant force from the acoustic pressure waves to be exerted upon the tympanic bulla at the interface with soft tissue [22]. Since the malleus is fused to the bulla, the force applied to the tympanic bulla will cause some motion in the ossicular chain, resulting in motion at the stapes footplate, which sits in the oval window of the cochlea. The pressure delivered to the surface of the tympanic bulla is the source of forcing for the ***pressure mechanism*** of sound reception.

The models that incorporate the ***pressure mechanism*** are essentially identical to those that we used to investigate sound reception in toothed whales [22, 33, 34]. That work led us to understand that the odontocete head works like an acoustic antenna; the entire surface of the animal’s head receives sound, and the anatomy channels the sound toward the ears. This head-as-an-acoustic-antenna hypothesis probably applies to all mysticetes too.

**Fig 3:**
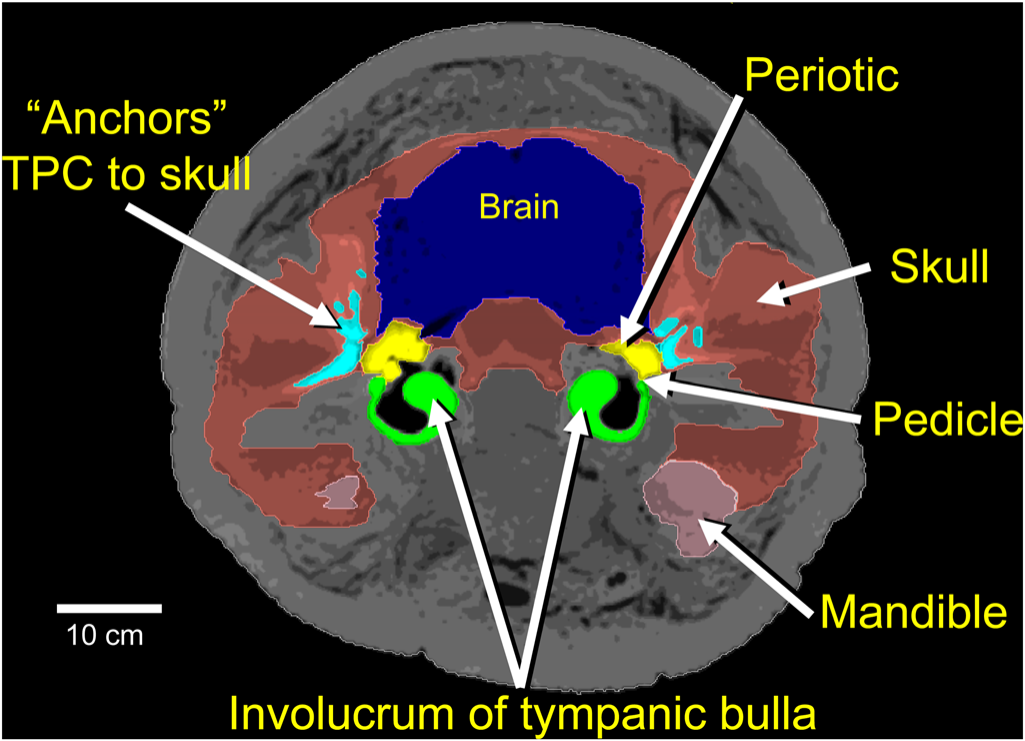
Transverse section through the otic region in the head of a fin whale calf. The bony projections that “anchor” the tympanoperiotic complexes to the skull are cyan. The brain is blue, the skull is salmon colored, and the mandible is pink. The periotic portions of the TPC are yellow, and each tympanic bulla is green. Note that thin bony pedicles form a fulcrum for differential vibration between the periotic bones and the large hypermineralized masses of the tympanic bulla at the distal end of each involucrum.

The ***bone conduction mechanism*** was discovered by observing skull deformations that are associated with the elastic waves propagating through the head. Skull vibrations are apparently transmitted through the bony anchors to the periotic portion of the TPC (Figs. 1 and 2). Each tympanic bulla forms a lever arm that hangs from two thin bony struts (pedicles) (Fig. 3), and ends in the massive thickened rim at the distal tympanic bone, known as the involucrum [54]. The tympanic bone essentially “swings” on the pedicles [23], which function as the fulcrum of the lever arm. The flexing occurs in response to the differential motion between the periotic bones and the tympanic bulla, enhanced by the inertial properties of the hypermineralized [55] involucrum, causing it to lag behind the motions of the skull and periotic. The malleus is fused to the tympanic bone in all extant cetaceans, so any motion of the tympanic bone is transmitted through the middle ear ossicles that push on the oval window of the inner ear.

This lever arm construction is common to all cetacean TPCs (Archaeoceti, Odontoceti, and Mysticeti) [17, 23, 56], and is perhaps the single most innovative adaptation that allowed underwater hearing to evolve. The tympanic bullae in cetaceans develops precocially, such that it reaches adult size and shape very early in life [57, 58], attesting to its essential incipient functional prominence. The primary difference across the cetaceans is the degree to which the periotic is attached to the rest of the skull, and may be an indication of the relative importance of ***bone-conduction*** versus ***pressure*** mechanisms in sound reception and transduction across all cetacean groups [23, 59, 60, 61].

For the skull-vibration induced bone conduction, the WP model yielded the amplitude of the displacements of the periotic bone and relative phase shifts between the components of displacement, while the FHV model simulated the response of the TPC to the forcing by prescribed harmonic-motion of the periotic bones.

For both the pressure and the bone conduction mechanisms, the result of the simulation was a transfer function (TF) between the amplitude of the incident sound pressure wave in the environment around the head and the magnitude of the velocity of the stapes footplate, the stapes-velocity transfer function (SVTF). Since two models were used to construct the SVTF for both loading mechanisms, the result is a composition of two TFs, WP-TF for the wave propagation model and FHV-TF for the forced vibration model.

***For the pressure mechanism***, the WP-TF describes the pressure acting on the tympanic bone as a function of the amplitude of the incident pressure wave, and the FHV-TF describes the velocity of the stapes footplate as a function of the pressure on the surface of the tympanic bone.

***For the bone conduction mechanism***, the WP-TF describes the motion of the periotic bone due to the vibration of the skull caused by a given amplitude of the incident pressure wave, and the FHV-TF describes the velocity of the stapes footplate as a function of the differential motions between the periotic bone and the bulla.

We note that Tubelli and colleagues [62] computed transfer functions for the minke whale middle ear that are analogous to the FHV-TF (for the pressure loading mechanism) in our two model series. Consequently, their results are only partial, as the wave propagation transfer function from the environment was not calculated. They considered two conjectural locations for application of loading by pressure, the tympanic bone and the tip of the glove finger. They did not consider the possibility of skull bone conduction.

**Fig 4:**
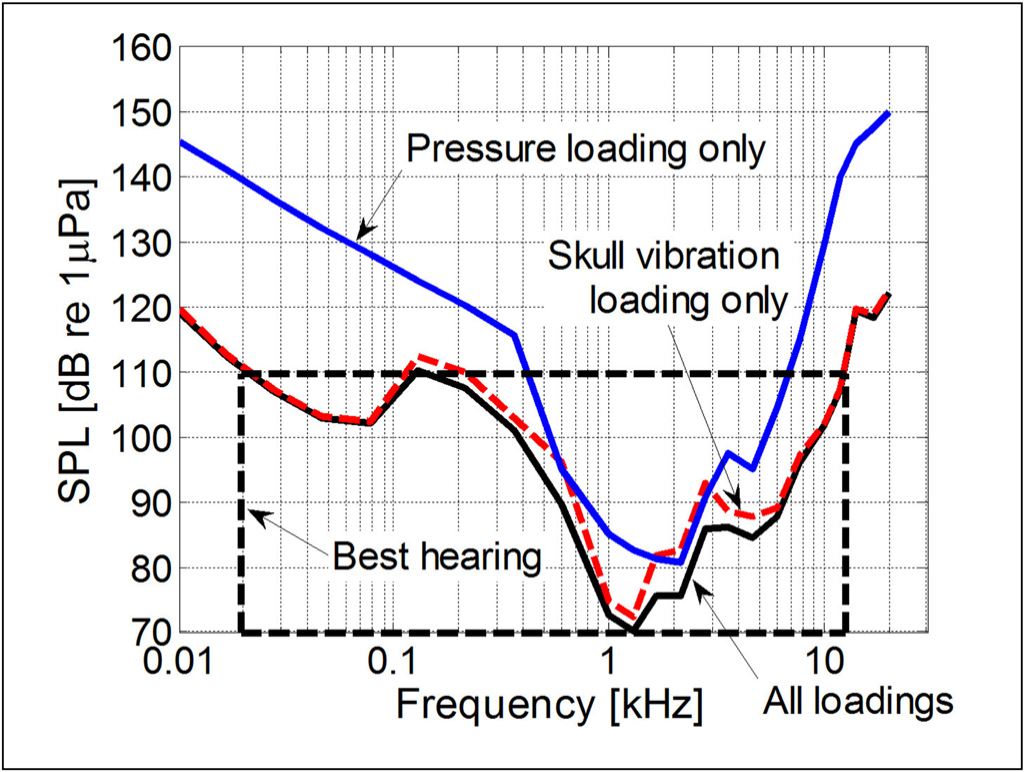
Predicted audiograms for the fin whale calf. The solid blue line represents the audiogram for the ***pressure mechanism***. The red dashed line represents the audiogram for the ***bone conduction mechanism***. The solid black line shows the combined audiograms for the ***pressure*** and ***bone conduction*** mechanisms.

With the SVTF at hand, we can attempt to predict the audiogram. The audiogram curve needs to be calibrated with the respect to the minimum audible pressure. Since this value has never been measured for a baleen whale, our approach was to set the hearing threshold to be similar to that measured for toothed whales, the bottlenose dolphin [41], or the killer whale [42], i.e. around 70 dB re 1 μPa at one meter. Then, the minimum threshold pressure across all frequencies can be estimated and the stapes velocity at the threshold can be consequently expressed through the maximum of the SVTF (see S1 File for a detailed explanation). Finally, the threshold pressure amplitude curve can be predicted from the SVTF as a function of frequency as shown in Fig. 4. The three curves correspond to the audiograms predicted from, (1) the pressure mechanism alone, (2) the bone conduction mechanism alone, and (3) from the sum of the effects of these two mechanisms.

Mysticete sound reception by the ***bone conduction mechanism*** is, according to Fig. 4, considerably more sensitive, particularly at low frequencies, than by the ***pressure mechanism***, indicating that the ***bone conduction*** mechanism is largely responsible for the mysticete whale’s sensitivity to low-frequency sound. Note that within the box (dashed lines) around the region of best hearing in Fig. 4, the curve drops by as much as 40 decibels, indicating better sensitivity in that range. Therefore, according to the synthetic audiograms generated by our finite element models (Fig. 4), the ***bone conduction*** audiogram is approximately four times more sensitive (lower threshold) between 1–2 kHz than the ***pressure*** audiogram. More significantly, Fig. 4 predicts that the difference in auditory sensitivity over the range of the lowest frequencies used by fin whales (10 Hz to 130 Hz), is between 10 to 30 dB (i.e. up to 10 times) more sensitive for the ***bone conduction*** mechanism than for the ***pressure*** mechanism.

This study uses the only currently available method capable of predicting relative sensitivities for sound reception in a mysticete over a broad range of frequencies, between 10 Hz and 12 kHz. Note that the lower frequencies (~20 Hz) propagate well in the ocean and are relatively less attenuated by the environment, so there may be no need for the best sensitivity to be located at those frequencies. The ***bone conduction mechanism*** produces the lowest thresholds (red dashed line in Fig. 4), when both mechanisms are considered in isolation. Therefore, the ***bone conduction mechanism*** is likely the dominant component in mysticete hearing.

## Discussion

Mysticete sound reception is enabled by the vibration of the relatively stiff and dense skull in response to the sound waves passing through the body of the whale. The advantage to mysticetes of using low-frequency (long-wavelength) sounds becomes evident when considering the motion or displacement of the scatterer (i.e. the skull), instead of the scattered pressure, as described by Rayleigh [63]. The scattered pressure from low-frequency acoustic waves becomes ineffective as an excitation mechanism, because the amplification of the scattered pressure on the surface of the TPC is negligible for waves longer than the body of the animal. Consider, for example, that the wave length for a 20 Hz sound in water is 75 m, which is at least three times longer than the bodies of largest fin whales [64]. At the same time, the amplitude of the oscillations (displacement) of the scatterer (skull) grows with the wavelength of the incident sound [65]. It is of interest to note that a similar vibration mechanism has been studied in fish, where the otoliths respond to long-wavelength sound by moving relative to the soft tissues attached to them [66].

The air spaces associated with the TPCs play a minor role for the pressure forcing mechanism, but only for high frequencies (above 5 kHz). At those frequencies, the air spaces helped to establish a “resonant cavity” for the sound waves propagating through the soft tissues towards the ears. The waves in the soft tissues are much too long below 5 kHz for the air spaces to be significant contributors to the pressure-distribution calculation. The most important function for these interconnected air spaces may be to maintain sufficient air volume in the tympanic cavity around the ossicular chain to allow the ossicles to vibrate free of damping or interference by nearby soft tissues. A similar mechanism has also been proposed for the enlarged pterygoid sinuses in *Ziphius cavirostris* [38].

Although these simulations were conducted with the skull geometry of a fin whale calf, the two basic mechanisms will not change significantly for the adult skull. The reasoning here is twofold. First, the tympanic bullae develop precocially [58]. Second, the firm connection between the TPC and the cranium is common to all mysticetes.

Adult fin whale skulls are approximately twice as long as they are wide [64], nearly the same length/width proportions as in our fin whale calf. According to True [64] the absolute dimensions of an adult fin whale skull are approximately four times longer and wider than our neonate skull. In order to understand the potential implication of adult skull size, we generated an artificial geometry by uniformly scaling the voxel dimensions by a factor of 4.0. This resulted in a skull of ~5 m in length, which we modeled with the WP model as for the neonate, but currently only for a 200 Hz incident sound. The displacement amplitudes of the periotic bone were slightly larger (1.1 to 2.0 times greater) than those calculated for the neonate at a similar frequency. Therefore, the WP+FHV model prediction for an adult is likely to result in an audiogram similar to those reported here for the neonate, and our preliminary result suggests that with an increase in skull size the frequency of best sensitivity may be shifted towards lower frequencies.

The audiogram is understood to be shaped by the external, middle, and inner ear connected in series [67]. Experimental data and models discussed therein point to the external and middle ear as the main contributors to low and mid frequency regions of the audiogram, and the cochlea is believed to contribute to the shape of the audiogram around the limit of high-frequency hearing. The graphs in Fig. 4 probably rise less sharply for the very high frequencies than would be observed experimentally, because in our transfer function we consider the effect of the cochlea only through a fluid load. Our predicted audiogram curves are certainly only approximations, but these approximations should be rather good for low and mid frequencies, until we begin to predict what happens at the high-frequency limit. For the fin whale, that high-frequency limit would probably begin around 10 kHz. We contend that our predictions are likely quite robust below 10 kHz, for the low and mid frequencies of concern with anthropogenic sound exposure.

Understanding the potential effects of anthropogenic noise on mysticetes is a subject that has long plagued U.S. regulatory agencies and concerned large-scale industrial users of the ocean environment. The results reported here provide a new tool for assessing these acoustic interactions.

## Supporting Information

S1 File This supporting information file contains a description of the modeling process and additional visualizations of the results, including animations, figures, and tables. (DOC)

S1 Fig Schematic of the two models that extract the cochlear input from the incident wave. (PNG)

S2 Fig (A) Finite element mesh 4, and (B) Close-up of the ossicular chain. (A) Finite element mesh 4 (approximately 41,000 nodes, 230,000 elements). The periotic bone is trimmed off, and the red markers at the top-right of the mesh indicate nodes with prescribed displacements (as dictated by the motion or the lack of the motion of the squamosal bone of the skull). (B) Close-up of the ossicular chain and the sigmoidal process in the foreground. The joints between the ossicles are shown in color: the annular ligament between the stapes and the oval window is yellow; the incudostapedial ligament is green, and a small portion of the malleoincudal ligament is blue (most of this ligament and the malleus are obscured by the sigmoidal process).
(TIF)

S3 Fig The damping ratio for the Rayleigh proportional damping model as a function of the frequency for the parameters shown in the text. (PNG)

S4 Fig Surface of the tympanic bone with applied damping condition to account for the interaction with the soft tissues during skull bone-conduction loading is indicated by dark red color. Mesh 4 as in S2 Fig.
(PNG)

S5 Fig Distribution of the total sound pressure in the head of the fin whale for a 4 kHz incident signal. Sound Pressure Levels (SPL) between -3 dB (pressure diminished with the respect to incident) and +6 dB (pressure amplified with respect to incident) are shown. The pressure is not displayed in the volume of the bones (gray regions) or in the volume of the air spaces or sinuses (black regions). (A) is a transverse section through the TPC with labels indicating the involucra (i) of the tympanic bulla and the expanded portion of the squamosal (sq); (B) is a coronal (horizontal) section through the TPC, at the level of the tympanic bullae (tb). Note the amplified pressure amplitude near the dorsal surface of the tympanic bullae (tb) from a reflection off of the squamosal bones (sq).
(PNG)

S6 Fig Transformation of the incident pressure to pressure at the TPC near the sigmoidal process (TPTF). Note that close to 1–2 kHz the incident pressure is magnified to arrive at the surface of the TPC almost doubled in amplitude.
(PNG)

S7 Fig Periotic-bone displacement transfer function (PDTF). (A) Amplitudes of the displacements, and (B) phase shift with respect to the dorsal-ventral displacement.
(TIF)

S8 Fig Stapes Velocity Transfer Function (SVTF) and approximate error. (A) SVTF(P) for two meshes: mesh 3, with 62,000 nodes, in solid line, and mesh 6, with 20,600 nodes, in dashed line. (B) Approximate error vs. the number of nodes in the model, where the smallest error is *E*_a,4_ = 0.061. The errors are for meshes 4,…,9 (right to left).
(TIF)

S9 Fig Deformations and motion of the skull for 100 Hz incident wave. Amplitude magnified 20,000 times. (Animated visualization link with displacements magnified by 20,000 times).
(GIF)

S10 Fig Deformations and motion of the skull for 250 Hz incident wave. Amplitude magnified 20,000 times. (Animated visualization link with displacements magnified by 20,000 times).
(GIF)

S11 Fig Deformations and motion of the skull for 1.0 kHz incident wave. Amplitude magnified 20,000 times. (Animated visualization link with displacements magnified by 20,000 times).
(GIF)

S12 Fig Deformations and motion of the skull for 2.0 kHz incident wave. Amplitude magnified 20,000 times. (Animated visualization link with displacements magnified by 20,000 times).
(GIF)

S13 Fig Motion of the TPC for pressure loading at 10 Hz. (Animated visualization link with displacements magnified by 5,000 times).
(GIF)

S14 Fig Motion of the TPC for pressure loading at 599 Hz. (Animated visualization link with displacements magnified by 5,000 times).
(GIF)

S15 Fig Motion of the TPC for pressure loading at 1 kHz. (Animated visualization link with displacements magnified by 5,000 times).
(GIF)

S16 Fig Motion of the TPC for pressure loading at 2.7 kHz. (Animated visualization link with displacements magnified by 5,000 times).
(GIF)

S17 Fig Motion of the TPC for pressure loading at 14.1 kHz. (Animated visualization link with displacements magnified by 5,000 times).
(GIF)

S18 Fig Motion of the TPC for skull-vibration loading at 129 Hz. (Animated visualization link with displacements magnified by 5,000 times).
(GIF)

S19 Fig Motion of the TPC for skull-vibration loading at 359 Hz. (Animated visualization link with displacements magnified by 5,000 times).
(GIF)

S20 Fig Motion of the TPC for skull-vibration loading at 1 kHz (Animated visualization link with displacements magnified by 5,000 times). (GIF)

S21 Fig Motion of the TPC for skull-vibration loading at 5.99 kHz (Animated visualization link with displacements magnified by 5,000 times). (GIF)

S22 Fig Motion of the TPC for skull-vibration loading at 20 kHz (Animated visualization link with displacements magnified by 5,000 times). (GIF)

S23 Fig Change of the SVTF(P) due to a change in the cochlear impedance. Dotted line: decrease by a factor of ½, Δ = 0.159; dashed line: increase by a factor of 2, Δ = 0.084.
(PNG)

S24 Fig Change of the SVTF(P) due to a change in the elastic modulus of the joints in the ossicular chain. Dotted line: decrease by a factor of ½, Δ = 0.65; dashed line: increase by a factor of 2, Δ = 0.85.
(PNG)

S25 Fig Change of the SVTF(P) due to a change in the Rayleigh system damping. Dotted line: decrease of ς_min_ by a factor of ½, Δ = 0.25; dashed line: increase of ς_min_ by a factor of 2, Δ = 0.24.
(PNG)

S26 Fig Change of the SVTF(P) due to a change in the Rayleigh system damping. Dotted line: decrease of *ω*_min_ by a factor of ½, Δ = 0.21; dashed line: increase of *ω*_min_ by a factor of 2, Δ = 0.22.
(PNG)

S27 Fig Change of the SVTF(U) due to a change in the surface impedance on the tympanic bone. Dotted line: decrease by a factor of ½, Δ = 0.139; dashed line: increase by a factor of 2, Δ = 0.144.
(PNG)

S28 Fig (A) The SVTF(P). (B) The audiogram predicted from the SVTF(P). (TIF)

S29 Fig (A) The SVTF(U). (B) The audiogram predicted from the SVTF(U). (TIF)

S1 Table Properties of materials used in the TPC simulations. (DOCX)

S2 Table Properties of materials used in the VATk simulations. (DOCX)

## References

[1] McDonald MA, Mesnick SL, Hildebrand JA (2006) Biogeographic characterization of blue whale song worldwide: using song to identify populations. J Cetacean Res Manage 8: 55–65.

[2] Ŝirović A, Williams LN, Kerosky SM, Wiggins SM, Hildebrand JA (2013) Temporal separation of two fin whale call types across the eastern North Pacific. Mar Biol 160: 47–57. 10.1007/s00227-012-2061-z 24391281 PMC3873066

[3] Tyack PL, Clark CW (2000) Communication and acoustic behavior of dolphins and whales. In: Au WWL, Popper AN, Fay RR, editors. Hearing by whales and dolphins. New York: Springer-Verlag pp. 156–224.

[4] Clark CW, Ellison WT (2004) Potential use of low-frequency sounds by baleen whales for probing the environment: Evidence from models and empirical measurements. In: Thomas JA, Moss CF, Vater M, editors. Echolocation in bats and dolphins. Chicago: The University of Chicago Press pp. 564–589.

[5] Hildebrand JA (2005) Impacts of Anthropogenic Sound. In: Reynolds JE III, Ragen TJ, Perrin WF, Reeves RR, Montgomery S, editors. Marine Mammal Research: Conservation Beyond Crisis. Washington, DC: The Johns Hopkins University Press pp. 101–134.

[6] Hildebrand JA (2009) Anthropogenic and natural sources of ambient noise in the ocean. Mar Ecol Prog Ser 395: 5–20. 10.3354/meps08353

[7] National Research Council (2003) Ocean Noise and Marine Mammals. Washington, D.C.: National Academies Press pp. 208. 25057640

[8] Castellote M, Clark CW, Lammers MO (2012) Acoustic and behavioral changes by fin whales (*Balaenoptera physalus*) in response to shipping and airgun noise. Biological Conservation 147: 115–122. 10.1016/j.biocon.2011.12.021

[9] Clark CW, Altman NS (2006) Acoustic detections of blue whale (*Balaenoptera musculus*) and fin whale (*B. physalus*) sounds during a SURTASS LFA exercise. IEEE Journal of Oceanic Engineering 31: 120–128. 10.1109/JOE.2006.872213

[10] Stokstad E (2014) U.S. regulators unveil new ocean noise rules for marine mammals. Science 343: 128 10.1126/science.343.6167.128 24408410

[11] Southall BL, Bowles AE, Ellison WT, Finneran JJ, Gentry RL, et al (2007) Marine mammal noise exposure criteria: Initial scientific recommendations. Aq Mam 33: 1–521.

[12] Clark CW (1990) Acoustic behavior of mysticete whales. In: Thomas JA, Kastelein RA, editors. Sensory Abilities of Cetaceans: Laboratory and Field Evidence. New York: Plenum Publishing Corporation pp. 571–583.

[13] Matthews JN, Rendall LE, Gordon JCD, Macdonald DW (1999) A review of frequency and time parameters of cetacean tonal calls. Bioacoustics 10: 47–71. 10.1080/09524622.1999.9753418

[14] Thompson TJ, Winn HE, Perkins PJ (1979) Mysticete sounds. In: Winn HE, Olla BL, editors. Behavior of marine animals: current perspectives in research. New York and London: Plenum Pulishing Coproration pp. 403–431.

[15] Watkins WA, Wartzok D (1985) Sensory biophysics of marine mammals. Mar Mammal Sci 1: 219–260. 10.1111/j.1748-7692.1985.tb00011.x

[16] Parks SE, Urazghildiiev I, Clark CW (2009) Variability in ambient noise levels and call parameters of North Atlantic right whales in three habitat areas. J Acoust Soc Am 125: 1230–1239. 10.1121/1.3050282 19206896

[17] Ketten DR (2000) Cetacean ears. In: Au WWL, Popper AN, Fay RR, editors. Hearing by whales and dolphins. New York: Springer-Verlag pp. 43–108.

[18] Ketten DR (1997) Structure and function in whale ears. Bioacoustics 8: 103–135. 10.1080/09524622.1997.9753356

[19] Ketten DR (1992) The cetacean ear: form, frequency, and evolution. In: Kastelein RA, Supin AY, Thomas JA, editors. Marine Mammal Sensory Systems. New York: Plenum Press pp. 53–75.

[20] Ketten DR (1994) Functional analysis of whale ears: adaptations for underwater hearing. IEEE Proc Underwater Acoust 1: 264–270.

[21] Yamato M, Ketten DR, Arruda J, Cramer S, Moore K (2012) The auditory anatomy of the minke whale (*Balaenoptera acutorostrata*): a potential fatty sound reception pathway in a baleen whale. Anat Rec 295: 991–998. 10.1002/ar.22459 PMC3488298 22488847

[22] Cranford TW, Krysl P, Amundin M (2010) A new acoustic portal into the odontocete ear and vibrational analysis of the tympanoperiotic complex. PLoS ONE: Public Library of Science. pp. e.0011927. 10.1371/journal.pone.0011927 PMC2915923 20694149

[23] Fleischer G (1978) Evolutionary principles of the mammalian middle ear. Advances in Anatomy Embryology and Cell Biology 55: 1–70. 10.1007/978-3-642-67143-2 735912

[24] Dahlheim ME, Ljungblad DK (1990) Preliminary hearing study on gray whales (*Eschrichtius robustus*) in the field. In: Thomas JA, Kastelein RA, editors. Sensory Abilities of Cetaceans: Laboratory and Field Evidence. New York: Plenum Publishing Corporation pp. 335–346.

[25] Clark CW, Clark JM (1980) Sound playback experiments with southern right whales (Eubalaena australis). Science 207: 663–665. 10.1126/science.207.4431.663 17749328

[26] Parks SE (2003) Response of North Atlantic right whales (*Eubalaena glacialis*) to playback of calls recorded from surface active groups in both the North and South Atlantic. Mar Mammal Sci 19: 563–580. 10.1111/j.1748-7692.2003.tb01321.x

[27] Croll DA, Clark CW, Calambokidis J, Ellison WT, Tershy BR (2001) Effect of anthropogenic low-frequency noise on the foraging ecology of Balaenoptera whales. Animal Conservation 4: 13–27. 10.1017/S1367943001001020

[28] Southall BL, Moretti D, Calambokidis J, DeRuiter SL, Tyack PL (2012) Marine Mammal Behavioral Response Studies in Southern California: Advances in Technology and Experimental Methods. Marine Technology Society Journal 46: 48–59. 10.4031/MTSJ.46.4.1

[29] Richardson WJ, Green CR Jr., Malme CI, Thomson DH (1995) Marine mammals and noise. San Diego: Academic Press 576 p.

[30] Krysl P, Cranford TW (2014) Directional hearing and Head-Related Transfer Function in the common dolphin (*Delphinus capensis*). In: Popper AN, Hawkins AD, editors. Effects of Noise on Aquatic Life II New York: Springer Science+Business Media, LLC.

[31] Cranford TW, Trijoulet V, Smith CR, Krysl P (2014) Validation of a vibroacoustic finite element model using bottlenose dolphin simulations: The dolphin biosonar beam is focused in stages. Bioacoustics 23: 161–194. 10.1080/09524622.2013.843061

[32] Oberrecht SP, Krysl P, Cranford TW (2014) Sound transmission validation and sensitivity studies in numerical models. In: Popper AN, Hawkins A, editors. Effects of Noise on Aquatic Life II New York: Springer Science+Business Media, LLC.

[33] Krysl P, Cranford TW, Wiggins SM, Hildebrand JA (2006) Simulating the effect of high-intensity sound on cetaceans: Modeling approach and a case study for Cuvier’s beaked whale (*Ziphius cavirostris*). J Acoust Soc Am 120: 2328–2339. 10.1121/1.2257988 17069328

[34] Cranford TW, Krysl P (2012) Acoustic function in the peripheral auditory system of Cuvier’s Beaked Whale (*Ziphius cavirostris*). In: Popper AN, Hawkins AD, editors. Effects of Noise on Aquatic Life. New York: Springer Science+Business Media, LLC pp. 69–72. 10.1007/978-1-4419-7311-5_15 22278452

[35] Krysl P, Hawkins AD, Schilt C, Cranford TW (2012) Angular Oscillation of Solid Scatterers in Response to Progressive Planar Acoustic Waves: Do Fish Otoliths Rock? PLoS ONE: Public Library of Science pp. e42591. 10.1371/journal.pone.0042591 PMC3415422 22912710

[36] Schilt CR, Cranford TW, Krysl P, Shadwick RE, Hawkins AD (2012) Vibration of the otoliths in a teleost. In: Popper AN, Hawkins AD, editors. Effects of Noise on Aquatic Life. New York: Springer Science+Business Media, LLC pp. 105–108. 10.1007/978-1-4419-7311-5_23 22278460

[37] Reeves RR, Stewart BS, Clapham PJ, Powell JA, Folkens PA (2002) Guide to Marine Mammals of the World. New York: Chanticleer Press 527 p.

[38] Cranford TW, McKenna MF, Soldevilla MS, Wiggins SM, Shadwick RE, et al (2008) Anatomic geometry of sound transmission and reception in Cuvier’s beaked whale (*Ziphius cavirostris*). Anat Rec 291: 353–378. 10.1002/ar.20652 18228579

[39] Ginsberg JH (2001) Mechanical and Structural Vibrations: Theory and Applications: Wiley.

[40] Homma K, Kim N, Puria S (2011) Towards Creation of a Human-head Auditory Model for Simulating Bone-Conduction Pathways. AIP Conference Proceedings 1403: 552–553. 10.1063/1.3658146

[41] Johnson CS (1968) Masked tonal thresholds in the bottlenosed porpoise. J Acoust Soc Am 44: 965–967. 10.1121/1.1911236 5683663

[42] Szymanski MD, Bain DE, Kiehl K, Pennington S, Wong S, et al (1999) Killer whale (*Orcinus orca*) hearing: Auditory brainstem response and behavioral audiograms. J Acoust Soc Am 106: 1134–1141. 10.1121/1.427121 10462816

[43] Lillie DG (1910) Observations on the anatomy and general biology of some members of the larger Cetacea. Proc Zool Soc Lond LXXIV: 769–792. 10.1111/j.1096-3642.1910.tb01916.x

[44] Nummela S, Thewissen JGM, Bajpai S, Hussain T, Kumar K (2007) Sound transmission in archaic and modern whales: anatomical adaptations for underwater hearing. Anat Rec 290: 716–733. 10.1002/ar.20528 17516434

[45] Dwight T (1872) Description of the *Balaenoptera musculus* in the possession of the Boston Society of Natural History: with remarks on the classification of fin whales. Boston: Boston Society of Natural History. Memoirs.

[46] Fraser FC, Purves PE (1960) Hearing in cetaceans: Evolution of the accessory air sacs and the structure and function of the outer and middle ear in recent cetaceans. Brit Mus (Nat Hist), Bull Zool 7: 1–140.

[47] Purves PE (1966) Anatomy and physiology of the outer and middle ear in cetaceans. In: Norris KS, editor. Whales, dolphins and porpoises. Berkeley: Univ. of Calif. Press pp. 320–380.

[48] Ekdale EG, Berta A, Deméré TA (2011) The Comparative Osteology of the Petrotympanic Complex (Ear Region) of Extant Baleen Whales (Cetacea: Mysticeti). PLoS ONE. 10.1371/journal.pone.0021311 PMC3120854 21731700

[49] Norris KS (1964) Some problems of echolocation in cetaceans. In: Tavolga WN, editor. Marine bio-acoustics. New York: Pergamon Press pp. 317–336.

[50] Norris KS (1968) The evolution of acoustic mechanisms in odontocete cetaceans. In: Drake ET, editor. Evolution and environment. New Haven: Yale University Press pp. 297–324.

[51] Norris KS (1969) The echolocation of marine mammals. In: Andersen HT, editor. The biology of marine mammals. New York: Academic Press pp. 391–423.

[52] Norris KS (1975) Cetacean biosonar: Part 1 — Anatomical and behavioral studies. In: Malins DC, Sargent JR, editors. Biochemical and biophysical perspectives in marine biology. New York: Academic Press pp. 215–234.

[53] Cranford TW, Krysl P, Hildebrand JA (2008) Acoustic pathways revealed: Simulated sound transmission and reception in Cuvier’s beaked whale (*Ziphius cavirostris*). Bioinsp Biomim 3: e016001. 10.1088/1748-3182/3/1/016001 18364560

[54] Mead JG, Fordyce RE (2009) The therian skull: a lexicon with emphasis on the odontocetes. Smithsonian Contrib Zool 627: 216.

[55] Li Z, Pasteris JD (2014) Chemistry of bone mineral, based on the hypermineralized rostrum of the beaked whale (*Mesoplodon densirostris*). American Mineralogist 99: 645–653. 10.2138/am.2014.4571 25484370 PMC4251810

[56] Kellogg R (1936) A Review of the Archaeoceti. Washington: Carnegie Institution of Washington 336 p.

[57] Lancaster WC (1990) The middle ear of the Archaeoceti. J Vert Paleon 10: 117–127. 10.1080/02724634.1990.10011795

[58] Lancaster WC, Ary WJ, Krysl P, Cranford TW (2015) Precocial Development within the Tympanoperiotic Complex in Cetaceans. Marine Mammal Science 31(1): 369–375. 10.1111/mms.12145

[59] Fleischer G (1980) Low-frequency receiver of the middle ear in mysticetes and odontocetes. In: Busnel RG, Fish JF, editors. Animal Sonar Systems. New York: Plenum Publishing Corporation pp. 891–893.

[60] Fleischer G (1980) Morphological adaptations of the sound conducting apparatus in echolocating mammals. In: Busnel RG, Fish JF, editors. Animal Sonar Systems. New York: Plenum Publishing Corporation pp. 895–898.

[61] Fleischer G (1976) Hearing in extinct cetaceans as determined by cochlear structure. J Paleo 50: 133–152.

[62] Tubelli AA, Zosuls A, Ketten DR, Yamato M, Mountain DC (2012) A prediction of the minke whale (*Balaenoptera acutorostrata*) middle-ear transfer function. J Acoust Soc Am 132: 3263–3272. 10.1121/1.4756950 23145610 PMC4109219

[63] Rayleigh JWSB (1896) The Theory of Sound, Volume 2 New York: Macmillan 504 p.

[64] True FW (1904) The whalebone whales of the western North Altantic compared with those occuring in Eurpoean waters with some observations on the species of the North Pacific. Washington: Smithsonian Institution.

[65] Hickling R, Wang NM (1966) Scattering of sound by a rigid movable sphere. J Acoust Soc Am 39: 276–279. 10.1121/1.1909887

[66] DeVries HL (1950) The mechanics of the labyrinth otoliths. Acta Oto-Laryngologica 38: 262–273. 10.3109/00016485009118384 14856657

[67] Ruggero MA, Temchin AN (2002) The roles of the external, middle, and inner ears in determining the bandwidth of hearing. PNAS 99: 13206–13210. 10.1073/pnas.202492699 12239353 PMC130611

