# Correction: Fin Whale Sound Reception Mechanisms: Skull Vibration Enables Low-Frequency Hearing

**DOI:** 10.1371/journal.pone.0122298

**Published:** 2015-03-23

**Authors:** The PLOS ONE Staff

There are multiple errors in the Funding statement. Please refer to the correct Funding statement here.

TWC received funding from Michael Weise and James Eckman, at the Office of Naval Research (N00014–12–1–0516); Frank Stone, Ernie Young, and Robert Gisiner at the Chief of Naval Operations Environmental Readiness Division (CNO N45) as well as Curtis Collins and John Joseph at the Naval Postgraduate School (N00244–10–1–0054). The funders had no role in study design, data collection and analysis, decision to publish, or preparation of the manuscript.

In addition, the third sentence of the third to last paragraph of the “Results” section is incorrect. The correct sentence for this section is.

“Since this value has never been measured for a baleen whale, our approach was to set the hearing threshold to be similar to that measured for toothed whales, the bottlenose dolphin [41], or the killer whale [42], i.e. around 70 dB re 1 μPa.”
