# Supplementary material for: Fin Whale Sound Reception Mechanisms: Skull Vibration Enables Low-Frequency Hearing
Source: PLoS One. 2015 Jan 29;10(1):e0116222. doi: 10.1371/journal.pone.0116222 (PMC4310601; doi:10.1371/journal.pone.0116222)
Supplement: S1 File — (DOC) [file pone.0116222.s001.doc]

**Supporting Information:**

## Modeling

#### Acquisition of anatomical data

On 20 November 2003 a male fin whale (*Balaenoptera physalus*) calf stranded alive on Sunset Beach in Orange County, California. Personnel from Sea World, San Diego and the California Marine Mammal Stranding Network attempted a rescue of this animal but it died during transport. This work was carried out in strict accordance with the Stranding Agreement, issued pursuant to Section 112(c) of the Marine Mammal Protection Act, between NOAA's National Marine Fisheries Service Southwest Region (NMFS-SWR) and SeaWorld San Diego (SeaWorld) (administrative reference number 151410SWR200900478:SMW).

The stranded whale was 550 cm long, weighed 1,165 kg, and was assigned a Field-ID (JEH520) by the Los Angeles County Museum of Natural History. The necropsy on the postcranial carcass was not performed by us, but was instead directed by Dr. Judy St. Leger and performed with additional personnel from SeaWorld San Diego and the Los Angeles County Museum of Natural History. The intact head was removed for further study and frozen within 24 hours of death. After sufficient time to allow for complete freezing, the head was placed inside a 48 inch diameter Sonotube, in which we attached four 2 inch plastic solid rods for image registration and density calibration, and filled the remainder of the tube with two-component polyurethane insulation foam. The custom container was then transported to Hill Air Force Base on 19 January 2004 where it was placed into an industrial CT scanner (ICT-1500). This process for CT scanning large specimens was developed years earlier and details have been reported elsewhere (Cranford, 1999; Cranford et al., 2008b). The CT data was processed into 965 transverse serial slices composed of 1.3 mm cubic voxels.

After CT scanning, the head was returned to a freezer until it was dissected on 21 August 2006. When the necropsy of the head was conducted, the tissue handling protocol was approved by the Graduate and Research Affairs, Institutional Animal Care and Use Committee at San Diego State University (APF#: 09-05-014B). Possession of the head was provided by a Letter of Authorization from the National Oceanic and Atmospheric Administration and the National Marine Fisheries Service Southwest Region (Administrative File: 151408SWR2013PROOOl).

The skull has since been prepared and accessioned into the Museum of Biodiversity, Department of Biology at San Diego State University, currently identified as specimen S-970.

#### Outline of computational approach

Consider a progressive planar harmonic wave in water with mass density $\rho$, speed of sound $c$, and angular frequency $\omega$. The acoustic wave, which for this paper we assume arrives from directly ahead of the animal, impinges upon the whale's head, partially reflects off the skin and partially propagates further in the blubber and muscle and bone as elastic waves. As waves scatter and propagate further inwards, the tissues of the animal are set in motion. Eventually a steady state is reached, with a pattern of harmonic elastic waves that interact with the tissues of the animal. In particular, these elastic waves generate motion within the tympanoperiotic complex (TPC) which houses the middle and inner ear: The differential displacements of the stapes in the oval window of the cochlea are driven by the ossicular chain, where the malleus is fused to the tympanic bone. The tympanic and periotic bones are connected through relatively flexible bony pedicles. The differential motion between the periotic and tympanic bones can drive the ossicular chain, resulting in piston-like and other complex motions at the stapes footplate.

The TPC can be modeled to within some approximation as a vibrating solid, for instance with a finite element discretization as in this work and as in (Tubelli et al., 2012). The problem of the so-called forced harmonic vibration analysis can be readily solved, and the result in terms of a transfer function that transforms the incident sound pressure to some measure of the input into the cochlea can be interpreted as an approximation of an audiogram. In order to exercise such a model we need to determine the forcing to be applied to the model of the TPC.

Error: Reference source not found **Figure** illustrates our approach. The incident acoustic wave of pressure amplitude $p_{\text{inc}}$ in the seawater surrounding the animal interacts with the tissues of the animal and generates traction loads on the surface of the TPC, which can, to a good approximation, be considered to be driven by the acoustic pressures of amplitude $p_{\text{TPC}}$. The TPC vibrates under the action of the loads $p_{\text{TPC}}$ and the result is the motion of the stapes footplate within the oval window to generate the velocity at the center of the footplate $v_{\text{st}}$. The resulting transfer function ($p_{\text{inc}}$to $v_{\text{st}}$), the Stapes Velocity Transfer Function (SVTF), is the concatenation of *two* transfer functions: the first transfer function produces the pressure on the surface of TPC given the amplitude of the incident sound wave; the second transfer function produces the velocity of the stapes footplate given the pressure on the surface of the TPC. Correspondingly, we need two models to calculate the two transfer functions.

As an alternative to this scenario, we could also consider the possibility of the ossicular chain being put into motion by loading of the TPC that is analogous to bone conduction in humans (Homma et al., 2011). Error: Reference source not found **Figure** can be replicated for such conditions by replacing $p_{\text{TPC}}$ with some measure of the motion of the periotic bone, which is firmly embedded in the skull. The tympanic bone would be forced to follow the periotic bone, which is set into motion by the vibration of the skull, thereby exposing the ossicles to differential displacements. This is very similar to Bekesy’s “skull bone conduction” (Puria et al., 2012).

Both ways of loading of the TPC, by pressure delivered through soft tissues and by “skull bone conduction”, are discussed in detail below.


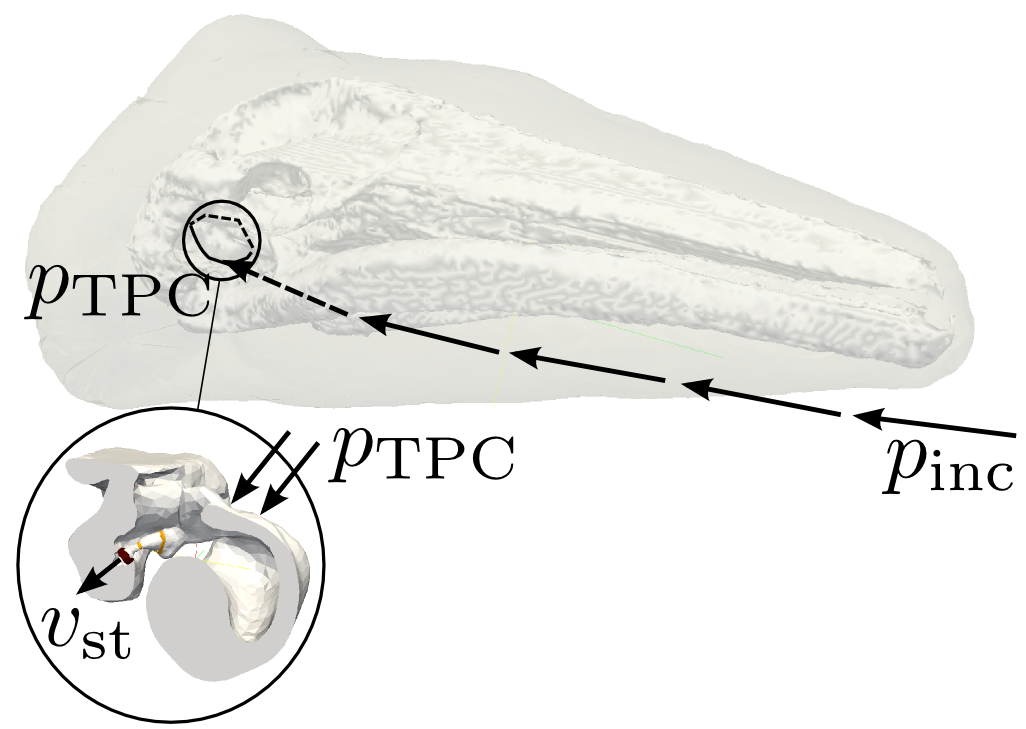


S1 Figure. Schematic of the two models that extract the cochlear input from the incident wave.

#### Harmonic vibration analysis

#### Equation of motion

The motion of the TPC was studied with a finite element model. The computational scheme was implemented in the Matlab toolkit FINEALE (Krysl, 2011). The system of (Newton’s) ordinary differential equations of motion was produced by the discretization in space with the finite elements as

(S1)

where the time-independent matrices are: $[K]$ the stiffness matrix, $[C]$ the damping matrix, and $[M]$ the mass matrix. The vector of applied harmonic loads is $[L_{\text{inc}}]=\exp(j\omega t)[\tilde{L}_{\text{inc}}]$, where the complex time-independent vector $[\tilde{L}_{\text{inc}}]$ describes the distribution of the loads and their phase shifts in time. The displacements of the nodes are consequently written with the same time dependence $[U]=\exp(j\omega t)[\tilde{U}]$, where the time independent vector of the unknown displacement amplitudes $[\tilde{U}]$ is again complex and encodes both the magnitude and the phase shift. After the time dependence is factored out we obtain the complex system of simultaneous algebraic equations

$-\omega^{2}[M][\tilde{U}]+j\omega[C][\tilde{U}]+[K][\tilde{U}]=[\tilde{L}_{\text{inc}}],$ (S2)

which need to be solved for the forcing frequency $\omega$ swept at discrete intervals through the frequency range of interest. Note that the time-independent vector $[\tilde{L}_{\text{inc}}]$ of the applied loads in general depends on the frequency. More details are given below.

#### Geometry and discretization in space

The geometry of the TPC was defined by a CT scan of the head of a juvenile fin whale with the dimension of the cubical voxels of 1.368 mm. The right-ear TPC volumetric image was processed as follows. The resolution of the CT image did not allow for the volume of the bone to be classified without user intervention: automatic thresholding procedure resulted in some parts of the ossicular chain to merge with the periotic bone, and the very thin parts of the tympanic bone were misclassified as soft tissue. Therefore, the CT scan was partially modified by hand and the thresholding procedure was based upon the best-guess augmentation of the automatic thresholding values. Furthermore, the resolution of the CT scan did not allow for an automatic detection of the ossicular chain joints and the stapedial annular ligament. The incudostapedial and incudomallear joints and the annular stapedial ligament were inserted manually into the thresholded volumetric images that then became the basis for the mesh generation.

The discretization by the finite element method in space was performed with tetrahedral elements and the nodal-integration technique as described in (Krysl et al., 2008a; Krysl et al., 2012a). The volumetric image with voxels classified either as empty space or as one of three materials, bone, joint ligament (incudostapedial and incudomallear), or annular stapedial ligament, was tiled with tetrahedra by subdividing each non-empty voxel into five tetrahedra. This initial mesh with 6.9 million tetrahedra was subsequently coarsened under constraints that enforced relatively fine mesh in the vicinity of the ossicular chain and the very thin parts of the tympanic bone and relatively coarse mesh elsewhere. A sequence of meshes from 1.4 million tetrahedra (mesh 1) to 36,000 tetrahedra (mesh 9) was obtained in this way. The sequence of meshes then could be used to assess the discretization error.


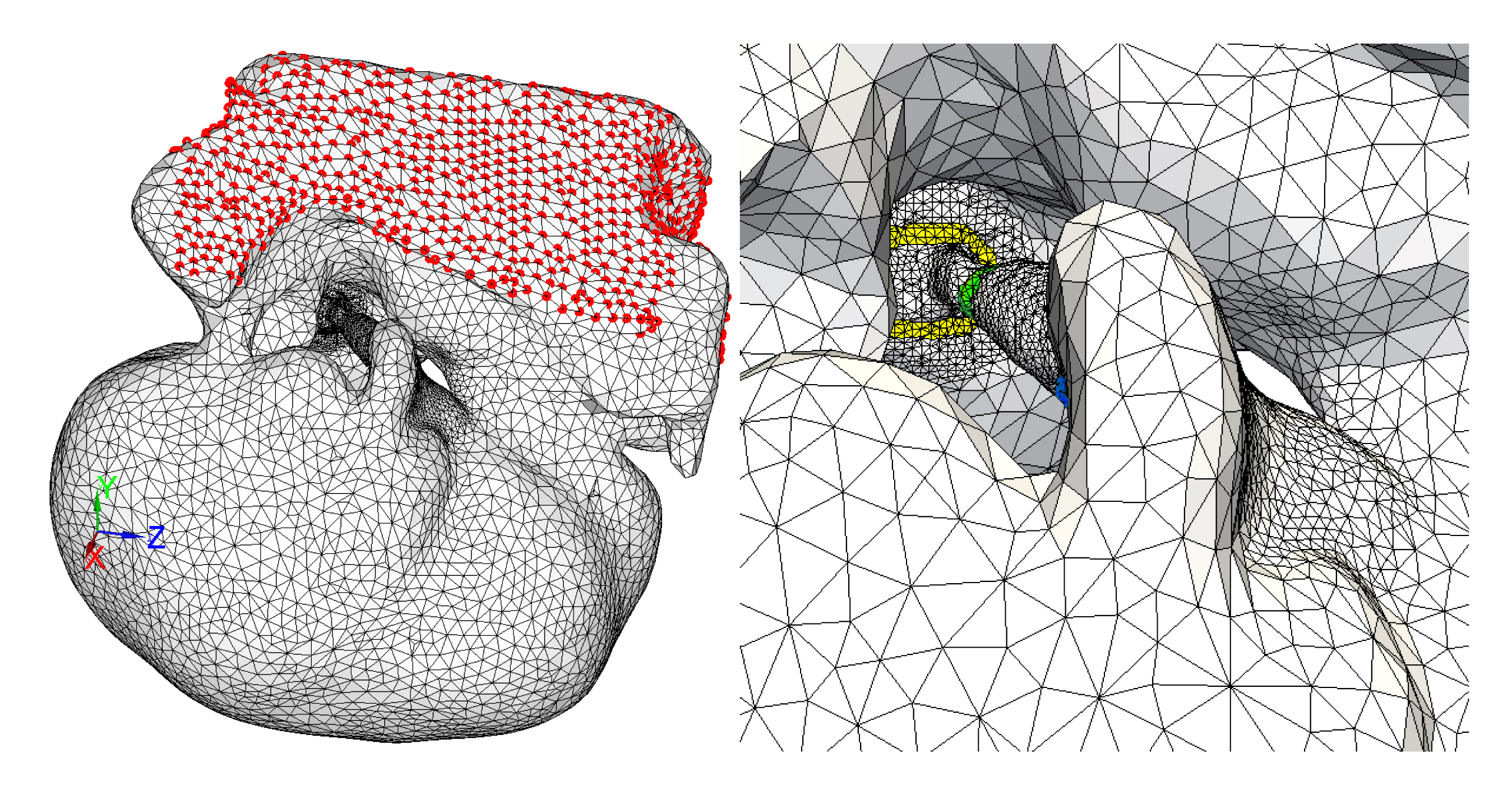


(A) (B)

S2 Figure. (A) Finite element mesh 4, and (B) Close-up of the ossicular chain. (A) Finite element mesh 4 (approximately 41,000 nodes, 230,000 elements). The periotic bone is trimmed off, and the red markers at the top-right of the mesh indicate nodes with prescribed displacements (as dictated by the motion or the lack of the motion of the squamosal bone of the skull). (B) Close-up of the ossicular chain and the sigmoidal process in the foreground. The joints between the ossicles are shown in color: the annular ligament between the stapes and the oval window is yellow; the incudostapedial ligament is green, and a small portion of the malleoincudal ligament is blue (most of this ligament and the malleus are obscured by the sigmoidal process).

#### Material properties

The material properties are summarized in Table Error: Reference source not found. The stiffness properties of the hard bone of the TPC our adopted from (Currey, 1979; Tubelli et al., 2012) and the mass density from (Nummela et al., 1999). Zhang and Gan have studied the properties of the incudostapedial joint in humans (Zhang et al., 2011). The joint ligament proved to be relatively soft and compressible. There do not seem to be any experimental measurements of the mechanical properties of the incudomallear joint in humans, but there appears to be agreement that at high frequencies the joint is flexible. Hence published modeling efforts include material properties for this joint (Cai et al., 2010; Volandri et al., 2012), but the scatter of the material properties is substantial. In the absence of a consensus, we adopt the elastic properties for both ossicular joints from (Cai et al., 2010) and (Zhang et al., 2011). Further, Gan et al. have studied the annular stapedial ligament elasticity in humans (Gan et al., 2011). As no other data are at this point available for this anatomical structure for the animal of interest, we adopt these estimates here as well. Homma et al. provide estimates of the mass density of the ligaments (Homma et al., 2009).

#### Damping

Both material damping and damping due to the cochlear fluid was considered. The damping matrix was therefore taken as

$[C]=[C_{m}]+[C_{c}]$ (S3)

where the first constituent matrix is the material damping matrix, and $[C_{c}]$ is the matrix due to cochlear-load damping.

The material damping was assumed in the form of the so-called Rayleigh proportional damping

$[C_{m}]=a_{s}[K]+a_{m}[M]$ (S4)

The parameters of the proportional damping $a_{s}$ and $a_{m}$ were determined from the condition that the damping ratio be bounded from below by $\varsigma_{\min}$ in the frequency range of interest. From the condition $\partial\varsigma/\partial\omega=0$ which is to hold at the angular frequency $\omega_{\min}$ we could determine the Rayleigh parameters as

$a_{s}=\varsigma_{\min}/\omega_{\min}$ and $a_{m}=\varsigma_{\min}\omega_{\min}$ (S5)

**Error: Reference source not found Figure** shows the damping ratio as a function of angular frequency. Evidently, this approach maintains the damping ratio reasonably close to the desired value around the central frequency, and the damping ratio increases towards the edges of the frequency range of interest. For the parameters $\varsigma_{\min}=0.025$, $\omega_{\min}=2\pi\times500 [rad]$ adopted for the fin whale TPC model the values were $a_{s}=7.96\times10^{-6} [\text{s}]$ and $a_{m}=78.5 [\text{s}^{\text{-1}}]$.

S3 Figure. The damping ratio for the Rayleigh proportional damping model as a function of the frequency for the parameters shown in the text.

The cochlear load was included by adding damping at the stapes footplate. The cochlear impedance of (Aibara et al., 2001), converted from RMS value, gives the resistive impedance constant of

$Z=\sqrt{2}\times21G\Omega\times A_{\text{FP}}\approx95500 [\text{Pa}\cdot\text{m}^{-1}\cdot\text{s].}$ (S6)

Finally, the damping matrix (S3) is also supplemented with another term for the bone-conduction simulations, see below and Error: Reference source not found **Figure**. The same type of damping as that for the cochlear load, but the impedance of seawater is used.

#### Loading of the TPC

#### Pressure loading

One possible source of forcing of the TPC are the tractions on its surface due to the elastic waves generated inside the whale’s head by the incident sound pressure. Since the shear part of the tractions is likely to be negligible due to the very low shear modulus of the soft tissues, we may simplify our argument by reducing the tractions on the surface of the TPC to the normal component, which in this case is approximately equal to the mean stress (the “sound pressure”) in the soft tissues next to the TPC.

#### Skull-motion loading

The second possible source of forcing of the TPC are the vibrations of the skull (Puria et al., 2012). The periotic bones are firmly attached to the skull by multiple bony processes, but the tympanic bones do not have a direct attachment to the skull. So, the skull bones are set into vibration by the scattering forces due to the impinging incident elastic waves in the soft tissues, from the incident sound pressure in the surrounding water. These skull vibrations will be carried to the periotic bone, and the tympanic bone will be forced to follow these vibrations through the connection to the periotic bone via the bony pedicles. Our model can tell us how much motion at the stapes footplate can be expected by this loading mechanism.

As the tympanic bone is also partially embedded in soft tissue, its presence must be accounted for in some way. In this work we adopt a simplified view of this interaction: the soft tissue is treated as water, and its effect is incorporated as a purely resistive force of damping by augmenting the damping matrix (S3). Error: Reference source not found **Figure** shows the surface on the tympanic bone to which the damping term is applied. The resistive impedance is adjusted to account for the observation that the soft tissue next to the vibrating tympanic bone is not naturally at rest, but rather in the absence of the hard bone would simply follow the same motion pattern as in the incident acoustic wave in the water. Therefore, for low frequencies the impedance should be decreased to reflect the nearly synchronous motion of the tympanic bone and the surrounding tissue, with impedance dropping to zero for vanishing frequency of the motion; on the other hand, for high frequencies with multiple wavelengths per the characteristic dimension of the tympanic bone, the pattern of the motions of the soft tissue means that the interaction in some places impedes the motion of the bone, in other places aids its motion by pushing in the same direction. Correspondingly, for very high frequencies we consider the impedance at full value. The impedance is thus ramped up from 0 kHz to full value at 2 kHz and then held constant.


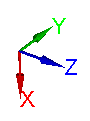

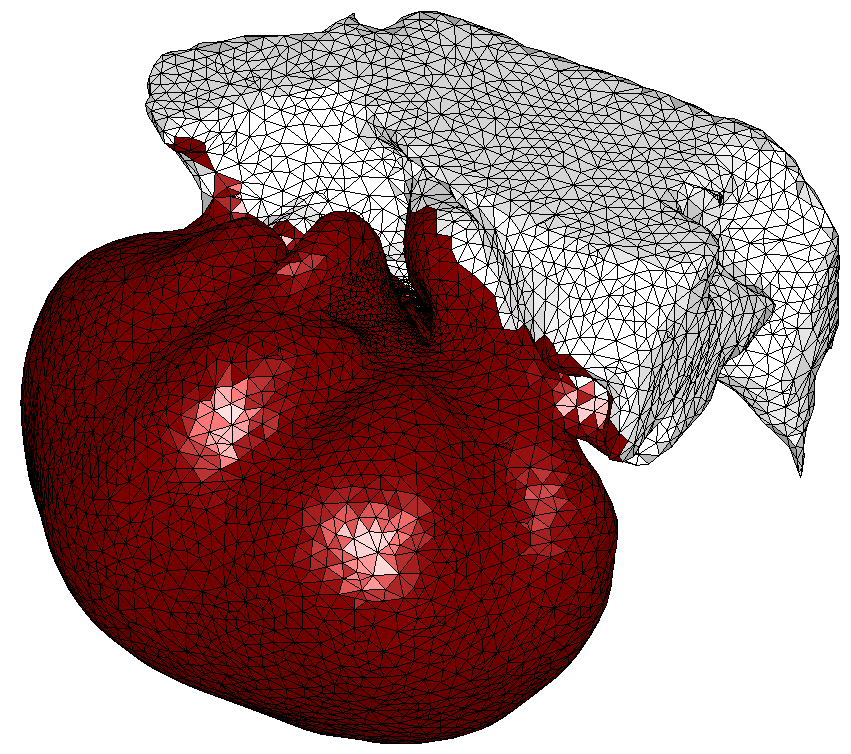


S4 Figure. Surface of the tympanic bone with applied damping condition to account for the interaction with the soft tissues during skull bone-conduction loading is indicated by dark red color. Mesh 4 as in S2 Figure.

#### Models of the loading of the TPC

#### Quantitative description of the forcing

Krysl and colleagues have previously introduced a finite element vibro-acoustic toolkit (VATk) for investigations of interactions of sound waves with a combination of solids, fluids, and cavities (Krysl et al., 2008b). This modeling approach has been recently validated with echolocation experimental data (Krysl et al., 2012b), and with experimental records of sound pressures within a bottlenose dolphin cadaver (Oberrecht et al., 2014). The technique has been previously applied in studies of the propagation of sound waves in marine mammals (Castellazzi et al., 2012; Cranford et al., 2012; Cranford et al., 2008a). The basic principle of this model is the superposition of the known incident displacement (pressure) field and the unknown perturbation displacement (pressure) field. The geometry of the volume of interest is a rectangular block divided into volumetric elements (voxels) of identical size and shape. The finite element method is used to discretize the geometry into elements that coincide with the voxels. The dynamics of the scattered waves is integrated in time with the centered difference method. The incident wave provides the forcing, and the scattered pressure wave is subject to absorbing boundary conditions at the boundary of the computational volume to only allow waves to leave. The absorbing boundary conditions in the present implementation are based on the plane-wave approximation.

#### Model for the forcing calculation

The geometry of the head of the fin whale was defined by CT scan, as described above. The voxels were cubic, 1.368 mm on each side. For simplicity the voxels were classified into one of only four material types: ear bone, skull, soft tissue which were taken as viscoelastic materials (refer to Table Error: Reference source not found the values of the material parameters; the loss factor of all materials was taken as 1/100), and seawater with mass density *ρ_w_*=1026 kg m^-3^ and sound speed *c_w=_* 1507 m s^-1^ .

In order to speed up the computations the initial CT scan was coarsened by a factor of three, and then converted to finite elements by mapping the voxel values to one of the material types introduced above. The resulting finite element model consisted of 205x190x324 or ~11.8 million cubic elements of 4.1 mm on a side. The incident sound wave was directed along the axis of the animal at frequencies 100, 250, 500, 1000, 2000, 4000, 8000, and 16000 Hz. At each of the excitation frequencies, after the steady-state vibration was reached, the amplitude of the total pressure in the soft tissues was extracted, as shown for the 4 kHz incident wave in Error: Reference source not found **Figure**. It is noteworthy that the reflections off the bony surfaces around the ear bones boost the pressure around the surface of the TPC above the 0 dB SPL, which represents the incident signal. This observation should be considered together with the recent investigation of the anatomy of the minke whale where a specialized fat body was described that filled the space around the thin part of the tympanic bone and eventually reached the ossicles in the vicinity of the sigmoidal process (Yamato et al., 2012). Our simulation results support the conjecture put forward in (Yamato et al., 2012) that these specialized fats play a role in delivering sound pressure to the surface of the TPC, and hence contribute to the function of the middle ear. In addition, we can see that reflective surfaces of the skull (ie., the lateral wing of the squamosal, see **S5 Figure** (B)) play a very important role, which may very well be the decisive factor that makes these acoustic fats useful components of the internal acoustic pinna.

The pressure waves through the air volume of the peribullary sinuses and other interconnected air cavities in this region were not modeled.


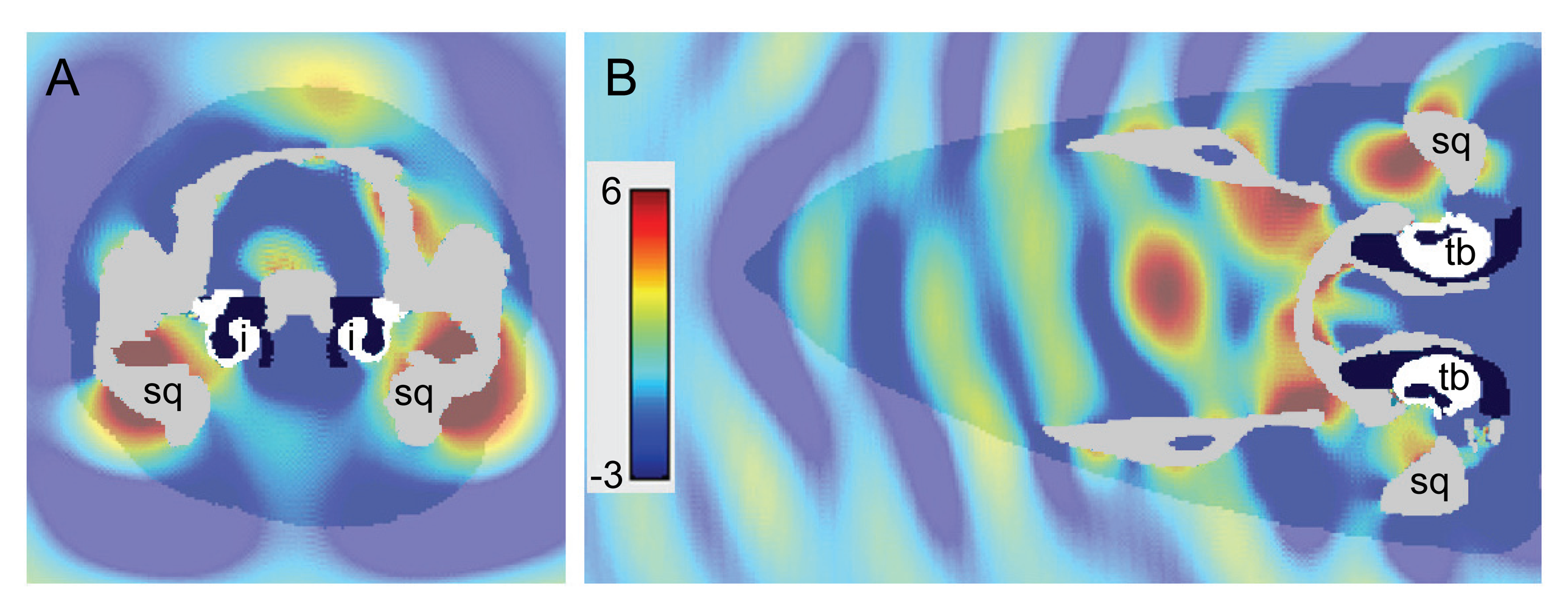


S5 Figure. Distribution of the total sound pressure in the head of the fin whale for a 4 kHz incident signal. Sound Pressure Levels (SPL) between -3 dB (pressure diminished with the respect to incident) and +6 dB (pressure amplified with respect to incident) are shown. The pressure is not displayed in the volume of the bones (gray regions) or in the volume of the air spaces or sinuses (black regions). (A) is a transverse section through the TPC with labels indicating the involucra (i) of the tympanic bulla and the expanded portion of the squamosal (sq); (B) is a coronal (horizontal) section through the TPC, at the level of the tympanic bullae (tb). Note the amplified pressure amplitude near the dorsal surface of the tympanic bullae (tb) from a reflection off of the squamosal bones (sq).

#### TPC Pressure Transfer Function

Using the model described in the previous section the TPC Pressure Transfer Function (TPTF) was calculated as shown in Error: Reference source not found **Figure**. Note that the value of TPTF above 1.0 means the incident pressure is amplified at the surface of the TPC; the value of the TPTF below 1.0 corresponds to attenuated amplitude.

S6 Figure. Transformation of the incident pressure to pressure at the TPC near the sigmoidal process (TPTF). Note that close to 1-2 kHz the incident pressure is magnified to arrive at the surface of the TPC almost doubled in amplitude.

#### Periotic Displacement Transfer Function

Our vibro-acoustic model delivers predictions of displacements throughout the computational volume as the primary solution variable. Therefore the model used above to predict the distribution of pressures at the same time provides estimates of the vibration amplitude of the skull bones. The periotic bone is firmly embedded in the skull, and as explained previously, the differential motion between the periotic bone and the tympanic bone may drive the ossicular chain. Error: Reference source not found **Figure** shows the Periotic-bone Displacement Transfer Function (PDTF). The amplitude of the plane-wave vibrations in the seawater corresponding to a frequency-independent incident pressure reads as follows

$u_{\text{inc}}=\frac{p_{\text{inc}}}{\rho_{w}c_{w}\omega}$ (S7)

and the PDTF is the ratio of the amplitude of the vibrations of the periotic bone along the Cartesian axes to $u_{\text{inc}}$.

(A) (B)

S7 Figure. Periotic-bone displacement transfer function (PDTF). (A) Amplitudes of the displacements, and (B) phase shift with respect to the dorsal-ventral displacement.

## Results

#### Stapes Velocity Transfer Function

The combination of the two models, the first to predict the pressure loading on the TPC, and the second to predict the stapes footplate velocity, results in the Stapes Velocity Transfer Function (SVTF). We will mark the SVTF obtained for the pressure loading on the tympanic bone with “(P)”, and the SVTF obtained for the skull-bone motion loading “(U)”.

Thus the pressure loading of the TPC results in the transfer function TF(P)

$TF(P)=\frac{v_{\text{st}}}{p_{\text{TPC}}}$ (S8)

and consequently the SVTF(P) is calculated as

$SVTF(P)=TF(P)\times TPTF=\frac{v_{\text{st}}}{p_{\text{TPC}}}\times\frac{p_{\text{TPC}}}{p_{\text{inc}}}$. (S9)

Analogously, for the skull-vibration loading of the TPC we obtain the transfer function TF(U) $TF(U)=\frac{v_{\text{st}}}{u_{\text{P}}}$ (S10)

and the SVTF(U) is calculated as

$SVTF(U)=TF(U)\times PDTF=\frac{v_{\text{st}}}{u_{\text{P}}}\times\frac{u_{\text{P}}}{u_{\text{inc}}}$. (S11)

#### Control of discretization error

For practical reasons the computational results in this study were obtained not with the finest mesh available, but with the mesh that gave reasonably accurate answers with acceptable computational cost. The mesh 3 with ~62,000 nodes was deemed sufficiently accurate as determined from the considerations of the approximate error of the *SVTF* calculated with mesh *j* defined as

$E_{\text{a},j}=\frac{\left\|SVTF_{j}-SVTF_{j-1}\right\|}{\left\|SVTF_{j-1}\right\|}$ (S12)

Here $E_{\text{a},j}$ is the normalized approximate error of the results computed with mesh *j*, $SVTF_{j}$ is the *SVTF* calculated with mesh *j*. Note that the meshes in the present study were obtained by coarsening, which means that mesh *j* is coarser than mesh *j-1*. Error: Reference source not found **Figure** shows two computed transfer functions for the finest mesh used and the mesh that is much coarser. The approximate error is also shown. It should be noted that the convergence rate of the transfer function measured in the above error is quite satisfactory as can be appreciated on the log-log plot.

(A) (B)

S8 Figure. Stapes Velocity Transfer Function (SVTF) and approximate error. (A) SVTF(P) for two meshes: mesh 3, with 62,000 nodes, in solid line, and mesh 6, with 20,600 nodes, in dashed line. (B) Approximate error vs. the number of nodes in the model, where the smallest error is $E_{\text{a},4}=0.061$. The errors are for meshes 4,…,9 (right to left).

The finite element mesh was deemed to provide sufficient accuracy when the change of the SVTF between the solutions for the previous mesh resulted in normalized approximate error smaller than 0.1.

#### Visualization of the skull deformations

The theory of scattering off of movable objects predicts ***increasing amplitude of motion for decreasing frequency of incident wave*** (of uniform amplitude). The skull motions generally follow these predictions, as shown in the following animation sequences. For 100 Hz incident wave the skull responds by very large amplitude of motion, which includes significant deformation of the lower jaw (Error: Reference source not found **Figure**; note that the deformations in **S9-S12 Figures** are magnified 20,000 times for clarity). For 250 Hz, Error: Reference source not found **Figure** shows gross skull and mandible deformations in addition to significant relative motion between the tympanic bone and the surrounding skull bones. For a 1.0 kHz incident signal, the deformations of the skull show finer detail (shorter-wavelength bending), and there is significant relative motion between the tympanic bone and the adjacent skull bones (Error: Reference source not found **Figure**). This trend continues in Error: Reference source not found **Figure** for a 2.0 kHz incident wave.


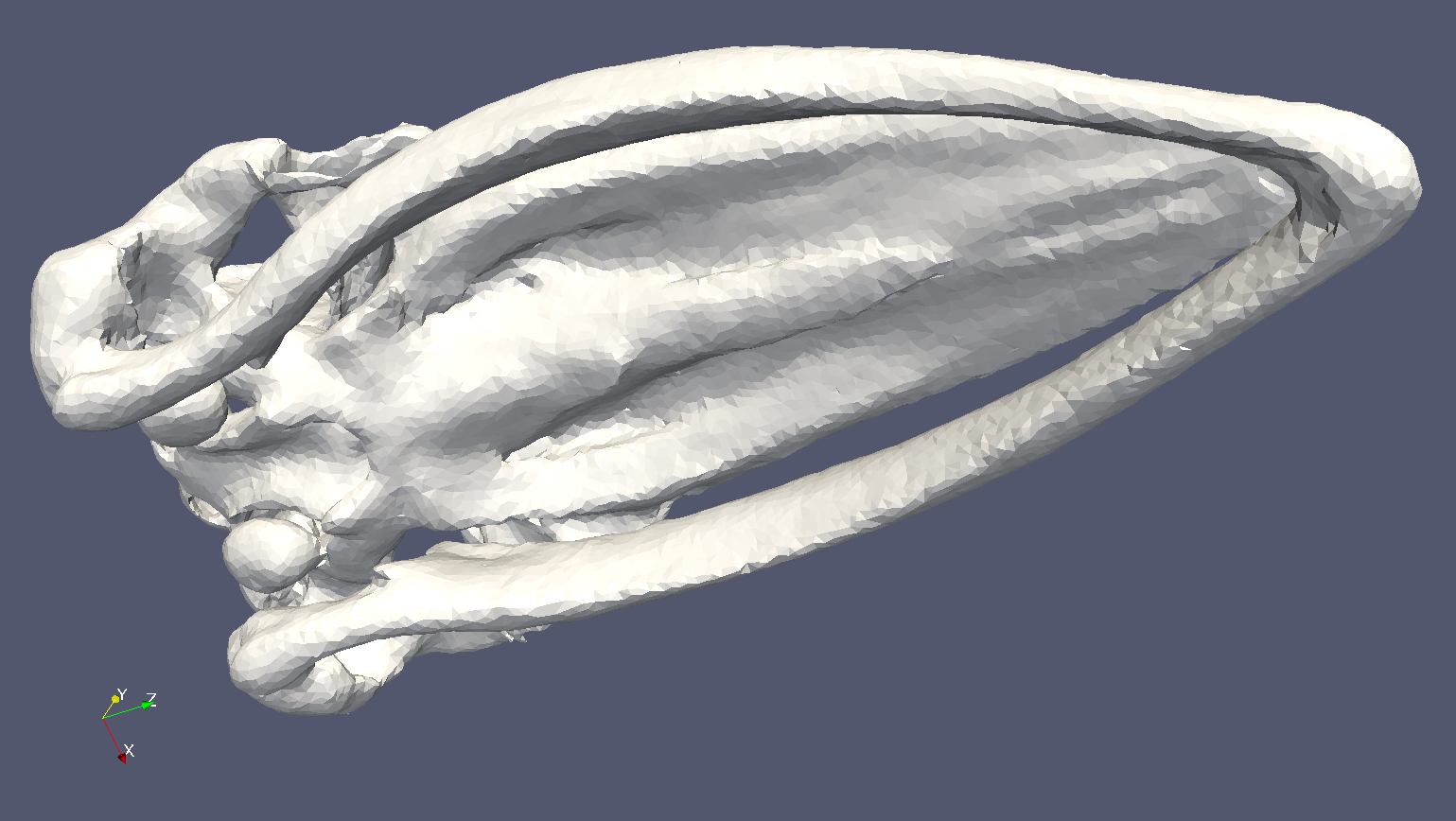


S9 Figure. Deformations and motion of the skull for 100 Hz incident wave. ([Animated visualization link with displacements magnified by 20,000 times](http://journals.plos.org/plosone/article/asset?unique&id=info:doi/10.1371/journal.pone.0116222.s010)).


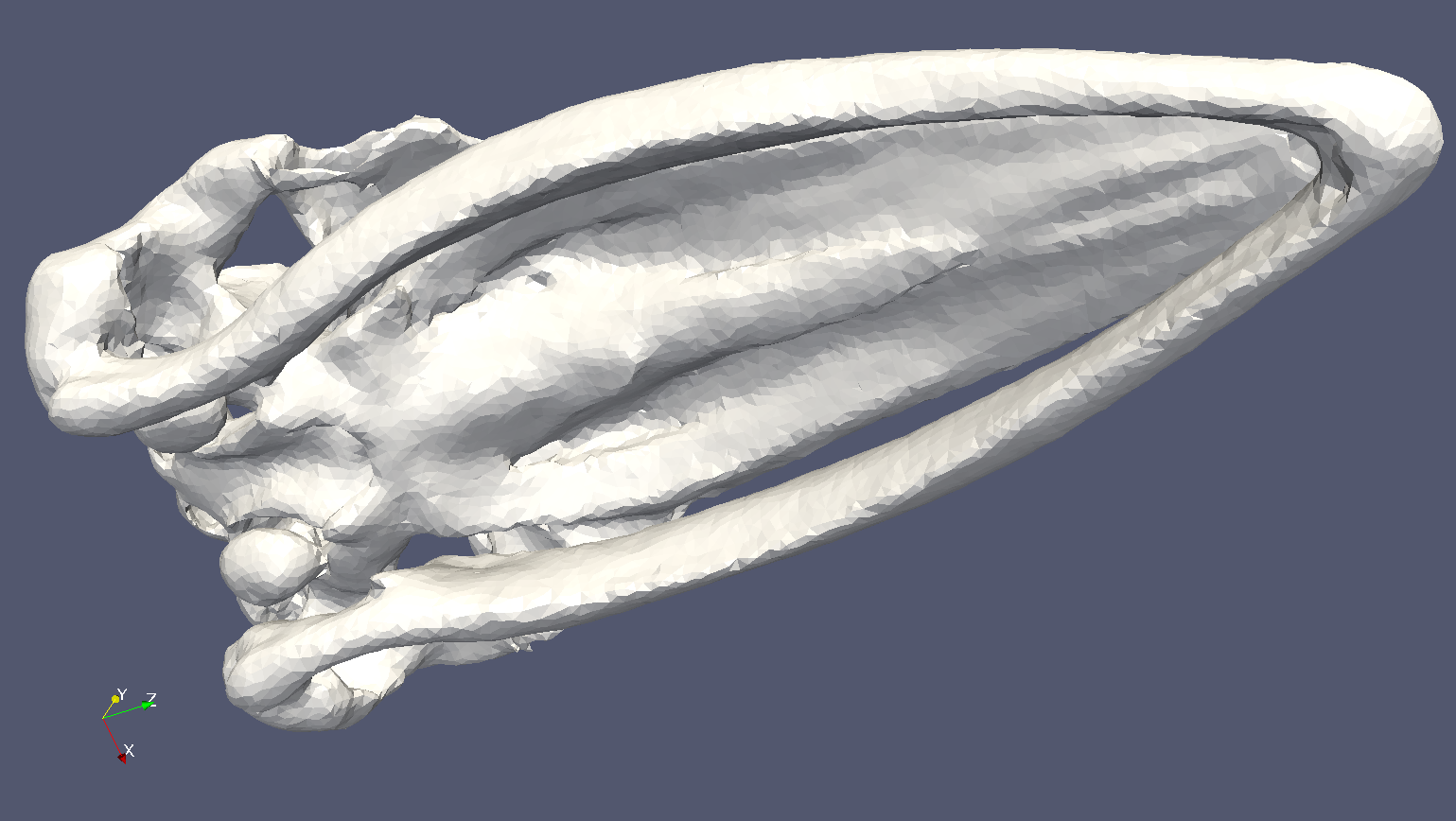


S10 Figure. Deformations and motion of the skull for 250 Hz incident wave. ([Animated visualization link with displacements magnified by 20,000 times](http://journals.plos.org/plosone/article/asset?unique&id=info:doi/10.1371/journal.pone.0116222.s011)).


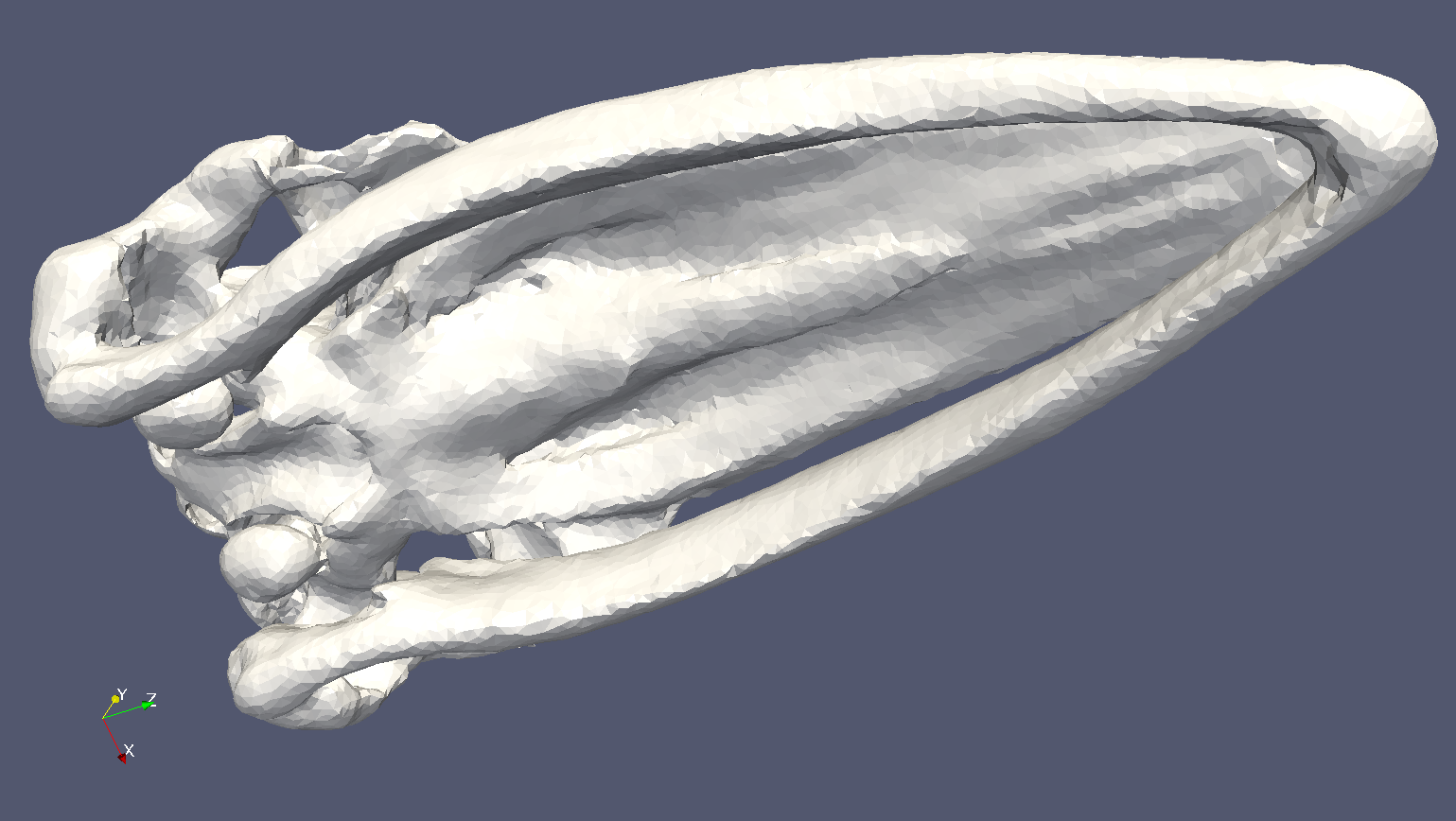


S11 Figure. Deformations and motion of the skull for 1.0 kHz incident wave. ([Animated visualization link with displacements magnified by 20,000 times](http://journals.plos.org/plosone/article/asset?unique&id=info:doi/10.1371/journal.pone.0116222.s012)).


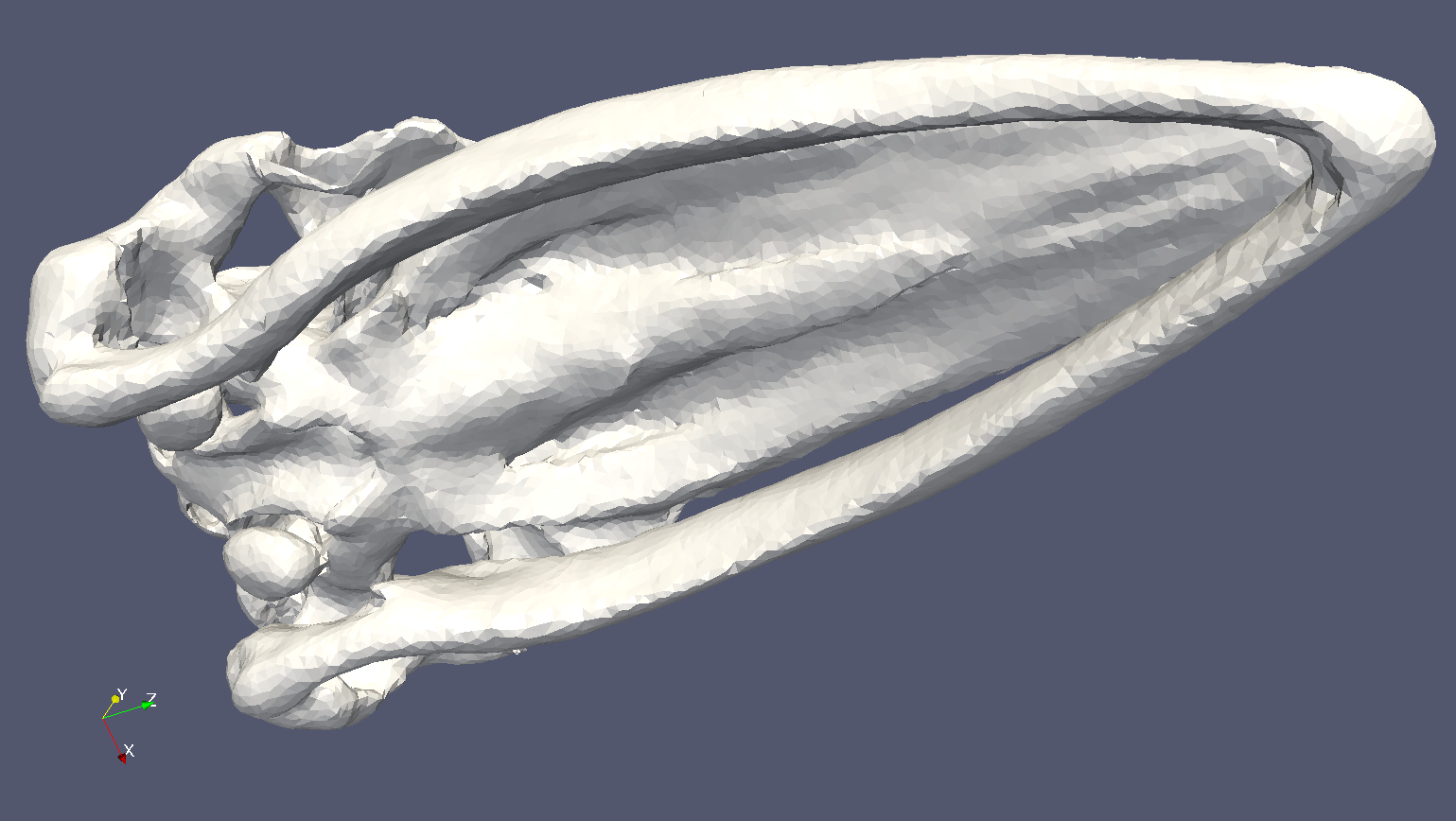


S12 Figure. Deformations and motion of the skull for 2.0 kHz incident wave. ([Animated visualization link with displacements magnified by 20,000 times](http://journals.plos.org/plosone/article/asset?unique&id=info:doi/10.1371/journal.pone.0116222.s013)).

#### Visualization of ossicular motion

In the figures that show the response of the TPC the color indicates the relative magnitude of the displacements. Blue corresponds to relatively small magnitude, while red corresponds to high displacement magnitude. Note that the motions of the animated shapes are highly exaggerated: displacements are magnified with a factor of 5x10^5^ for the pressure loading, and a factor of 1x10^4^ for the skull-vibration loading.

#### Pressure loading

**Error: Reference source not found Figure** shows the motion of the TPC due to pressure loading at 10 Hz. It can be described as pendulum-like and it is representative of motions up to roughly 130 Hz. For vibration shapes close to and above 200 Hz, one can detect slight motions of the ossicles themselves. At around 600 Hz, in addition to the pendulum-like swinging, the motion includes pronounced oscillation of the ossicular chain (Error: Reference source not found **Figure**). At 1 kHz the pendulum-like swinging is absent, and one can detect (apparently) resonant oscillation of the ossicles (Error: Reference source not found **Figure**). At approximately 2.7 kHz in addition to the vibration of the ossicular chain, the thin plate of the tympanic bone also begins to show localized bending deformation (Error: Reference source not found **Figure**). These patterns persist to around 8 kHz. At frequencies higher than 8 kHz, the pronounced motion of the ossicles can no longer be detected, and the bending of the tympanic plate becomes the dominant deformation pattern (Error: Reference source not found **Figure**).


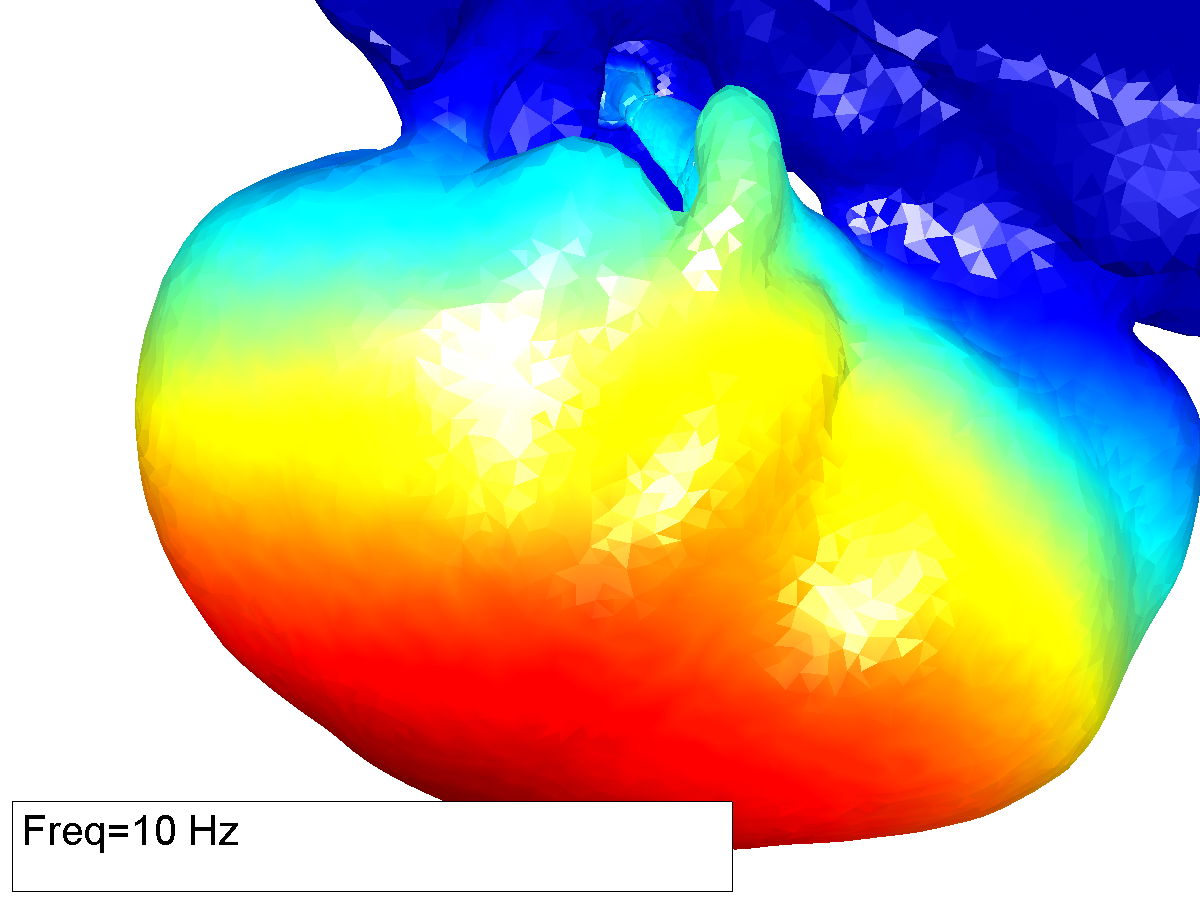


S13 Figure. Motion of the TPC for pressure loading at 10 Hz. ([Animated visualization link with displacements magnified by 5000 times](http://journals.plos.org/plosone/article/asset?unique&id=info:doi/10.1371/journal.pone.0116222.s014)).


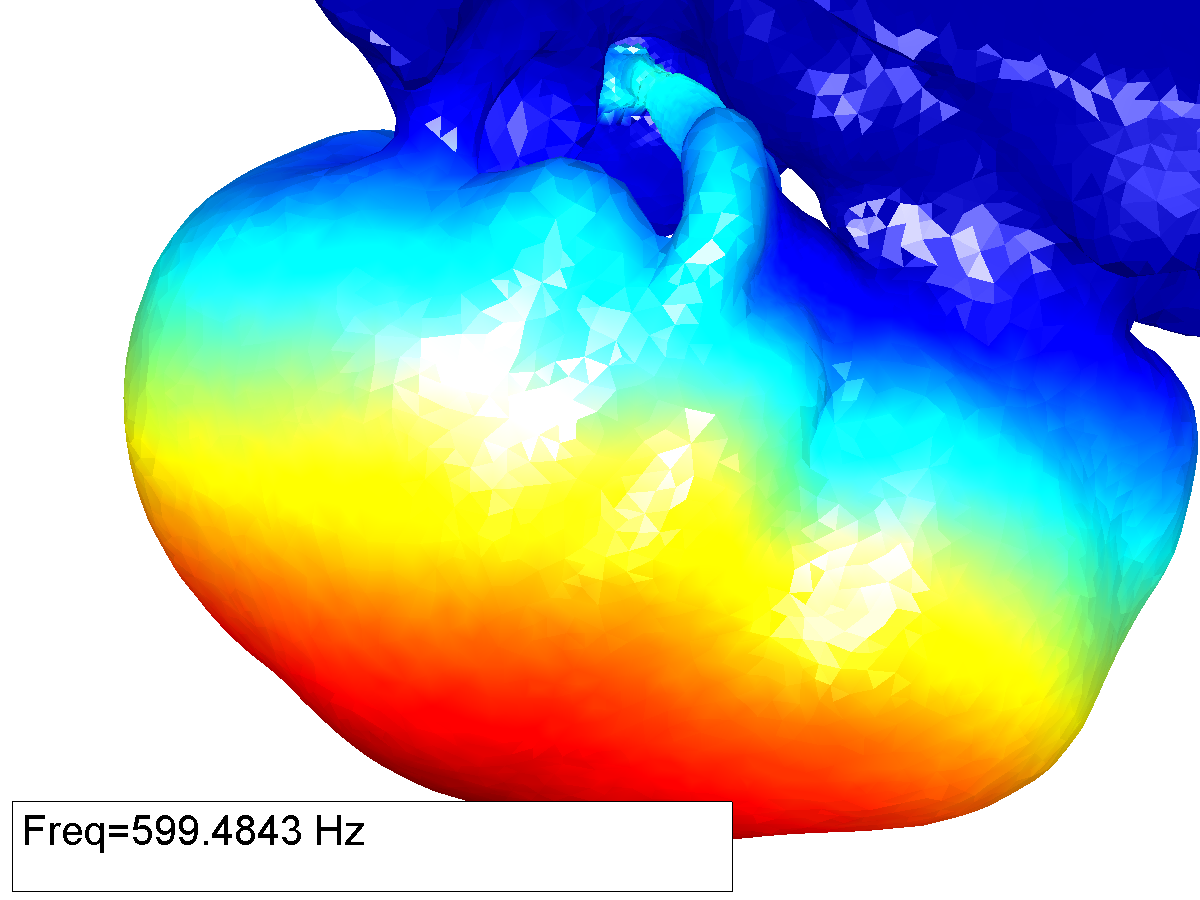


S14 Figure. Motion of the TPC for pressure loading at 599 Hz. ([Animated visualization link with displacements magnified by 5000 times](http://journals.plos.org/plosone/article/asset?unique&id=info:doi/10.1371/journal.pone.0116222.s015)).


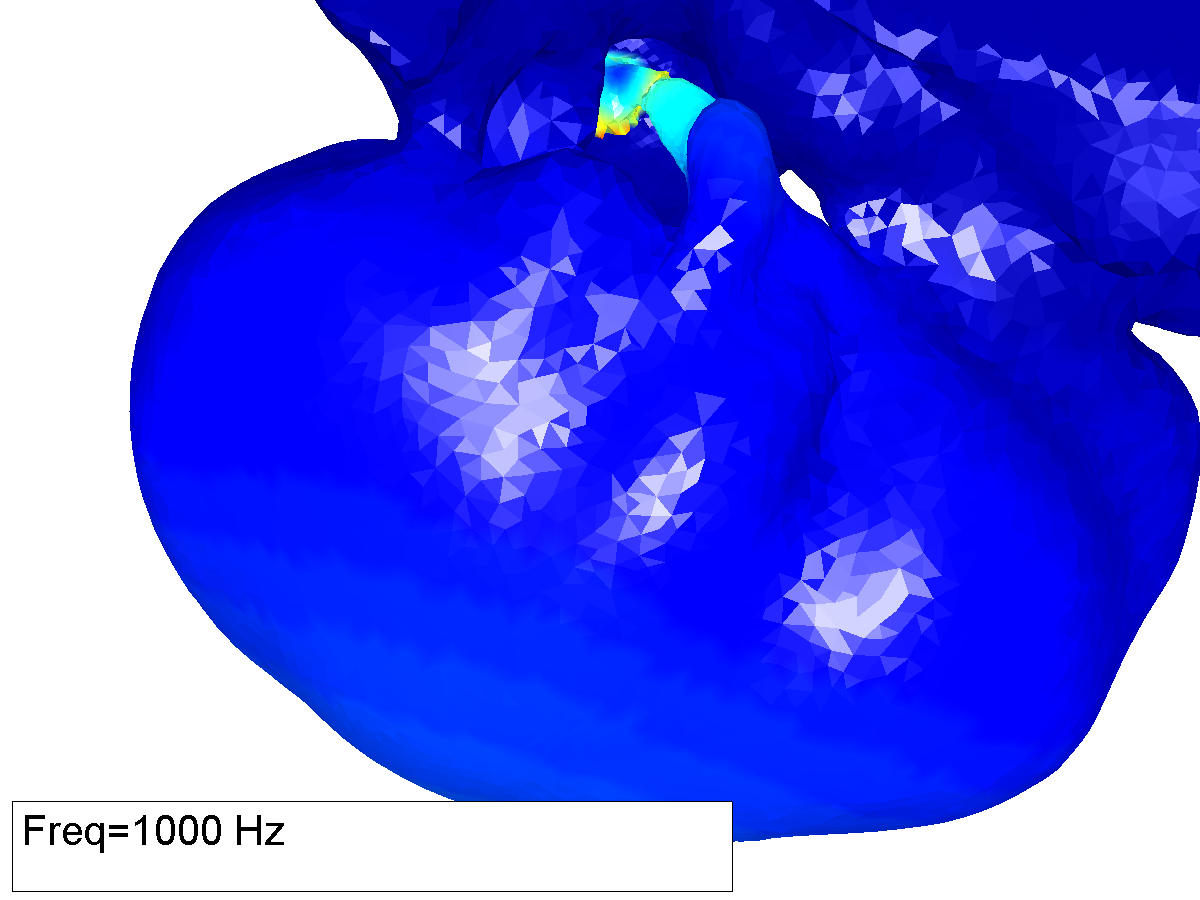


S15 Figure. Motion of the TPC for pressure loading at 1 kHz. ([Animated visualization link with displacements magnified by 5000 times](http://journals.plos.org/plosone/article/asset?unique&id=info:doi/10.1371/journal.pone.0116222.s016)).


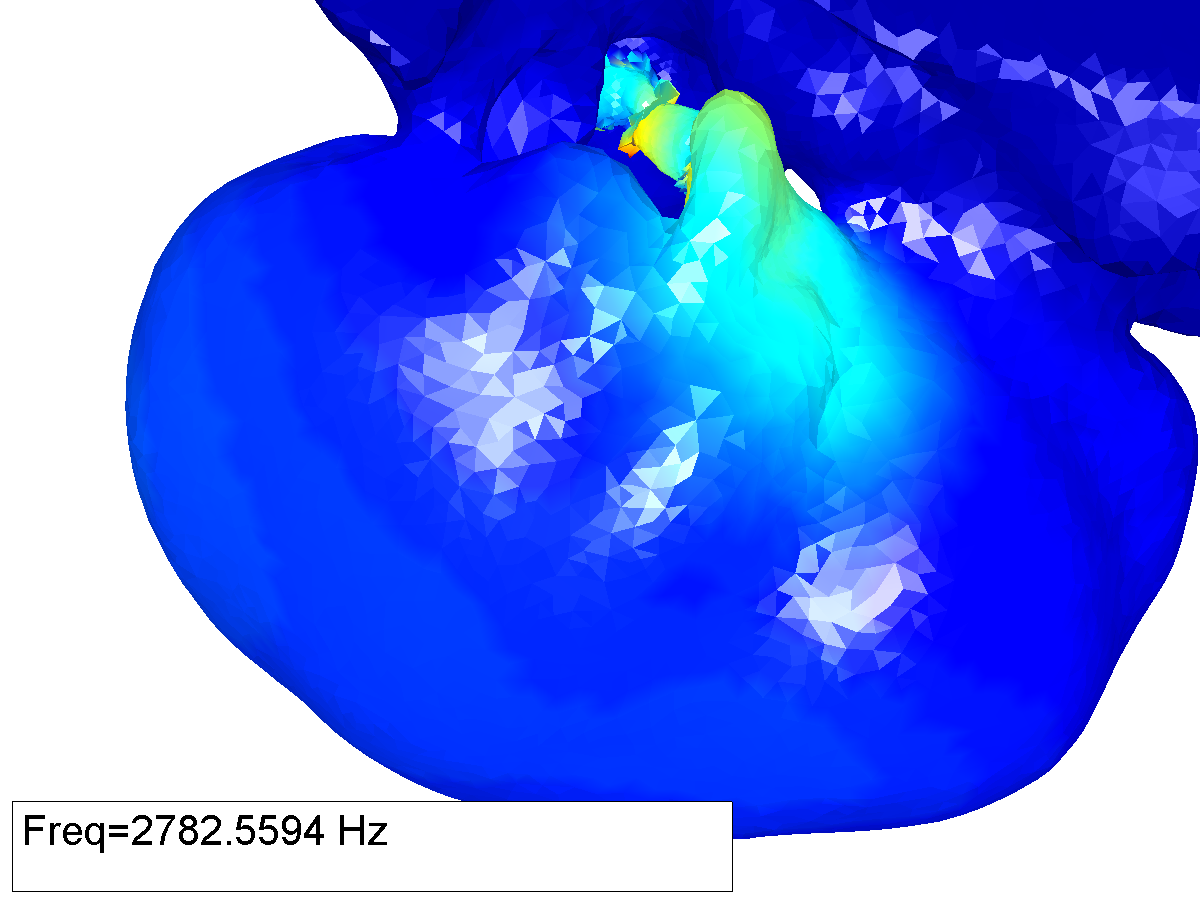


S16 Figure. Motion of the TPC for pressure loading at 2.7 kHz. ([Animated visualization link with displacements magnified by 5000 times](http://journals.plos.org/plosone/article/asset?unique&id=info:doi/10.1371/journal.pone.0116222.s017)).


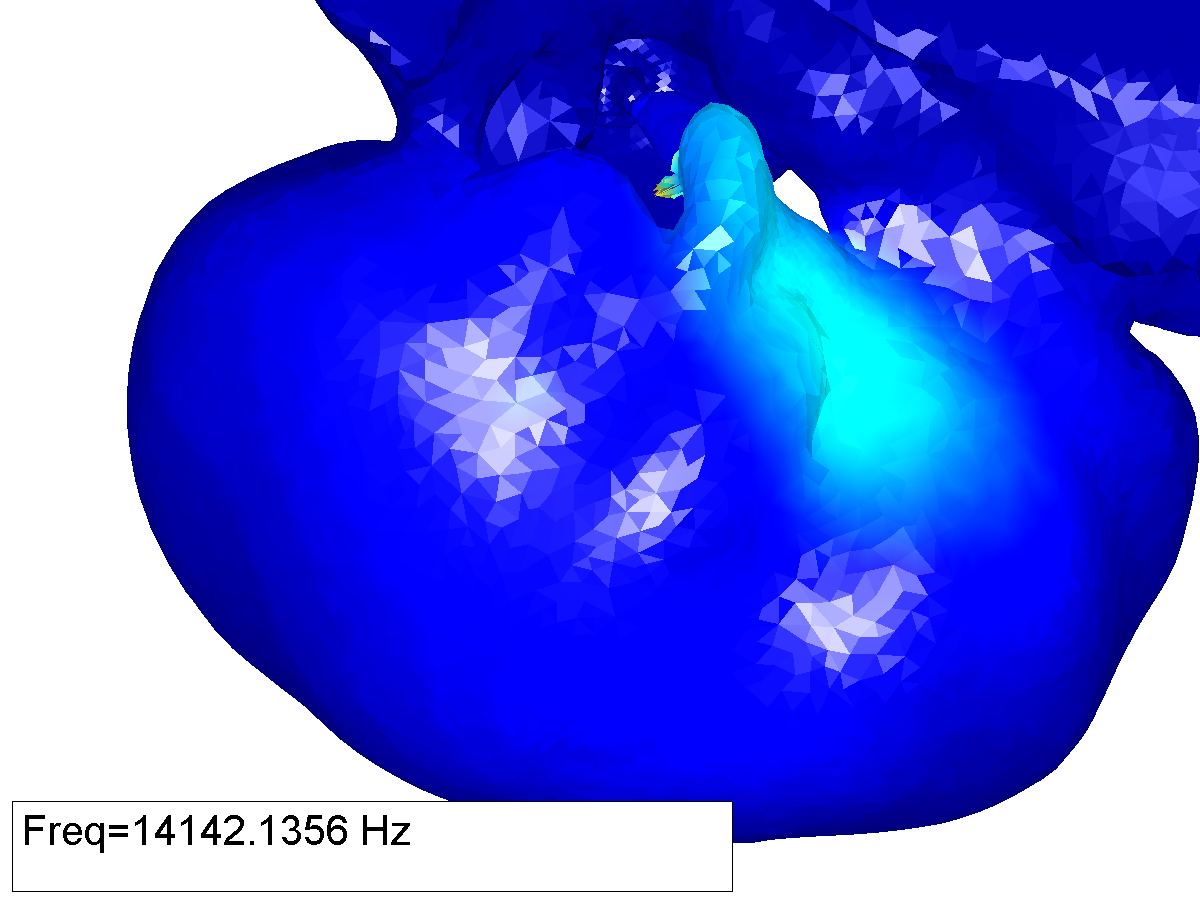


S17 Figure. Motion of the TPC for pressure loading at 14.1 kHz. ([Animated visualization link with displacements magnified by 5000 times](http://journals.plos.org/plosone/article/asset?unique&id=info:doi/10.1371/journal.pone.0116222.s018)).

#### Skull vibration loading

The visualized skull motions are of much larger amplitude than for the pressure loading: here the periotic bone is not fixed as for the pressure loading, but rather follows the relatively large oscillations of the skull. For low frequencies, the TPC swings pendulum-like as for low-frequency pressure loading. In Error: Reference source not found **Figure** (129 Hz) one can detect distinct activity of the ossicles leading to stapes motion. This becomes especially pronounced at 359 Hz (Error: Reference source not found **Figure**). At 1 kHz the pendulum-like swinging is reduced, and similarly to the pressure loading the (apparently) resonant oscillation of the ossicles becomes visually dominant (Error: Reference source not found **Figure**). At around 6 kHz the massive tympanic bulla begins to significantly lag behind the periotic bone. Error: Reference source not found **Figure** shows the displacement of the periotic bone relative to an almost stationary tympanic bone may be the source of stapes displacement. For 20 kHz the tympanic bone has become almost entirely stationary relative to the periotic bone (Error: Reference source not found **Figure**).


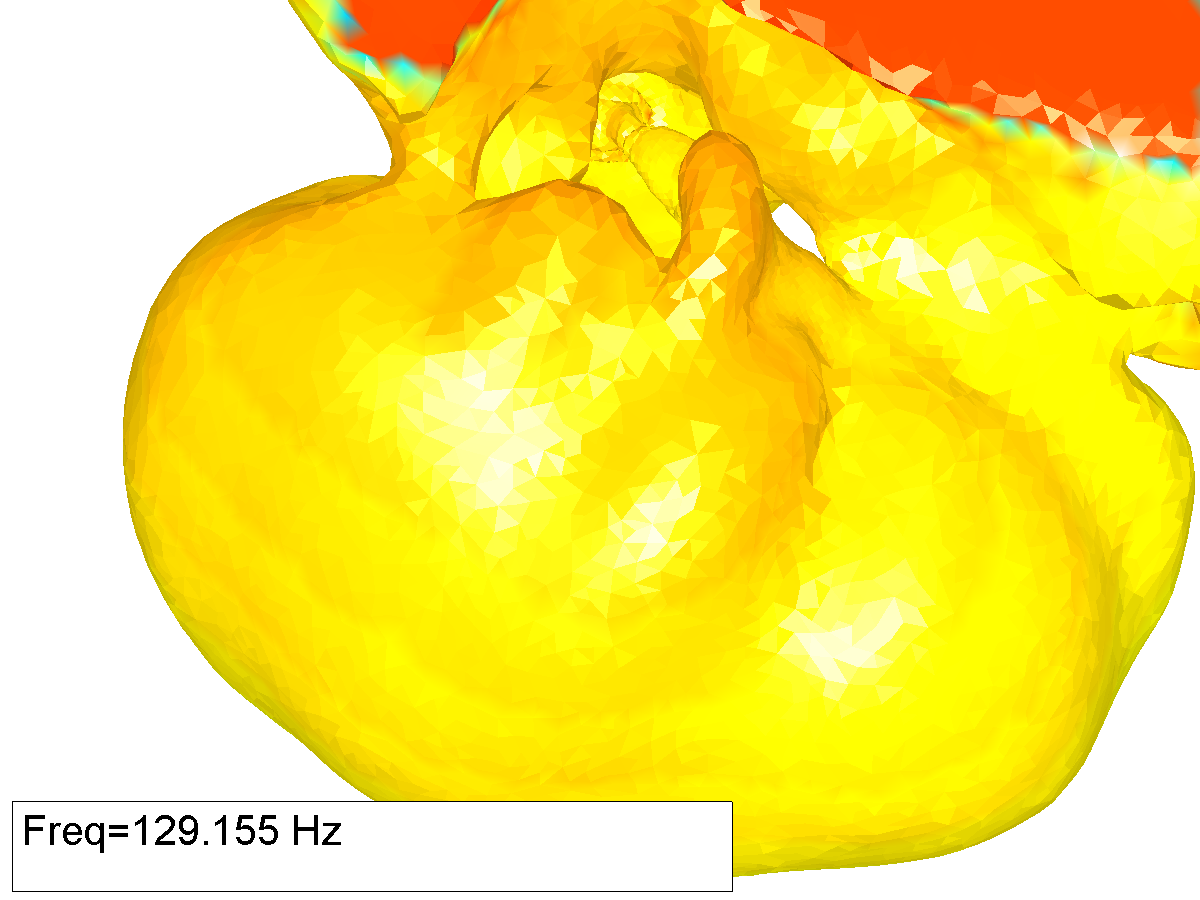


S18 Figure. Motion of the TPC for skull-vibration loading at 129 Hz. ([Animated visualization link with displacements magnified by 5000 times](http://journals.plos.org/plosone/article/asset?unique&id=info:doi/10.1371/journal.pone.0116222.s019)).


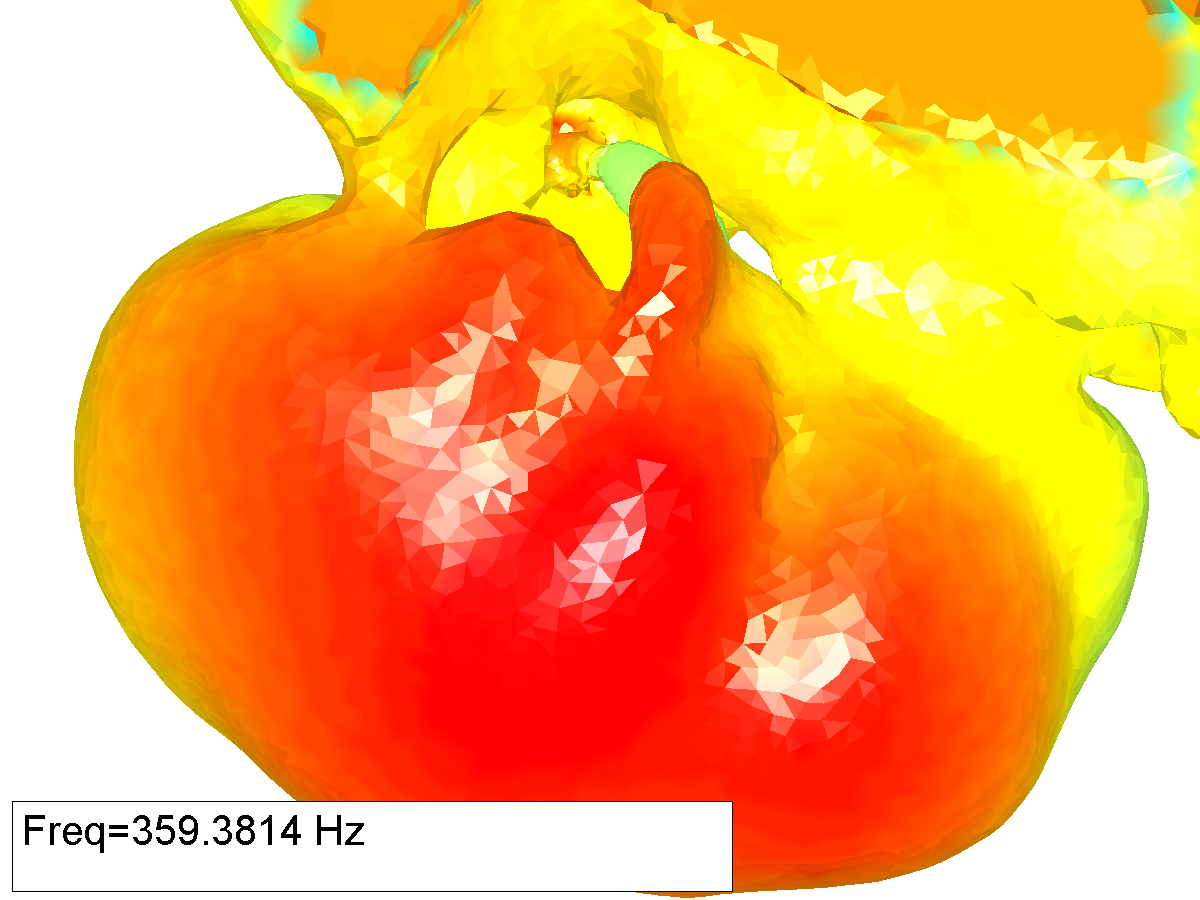


S19 Figure. Motion of the TPC for skull-vibration loading at 359 Hz. ([Animated visualization link with displacements magnified by 5000 times](http://journals.plos.org/plosone/article/asset?unique&id=info:doi/10.1371/journal.pone.0116222.s020)).


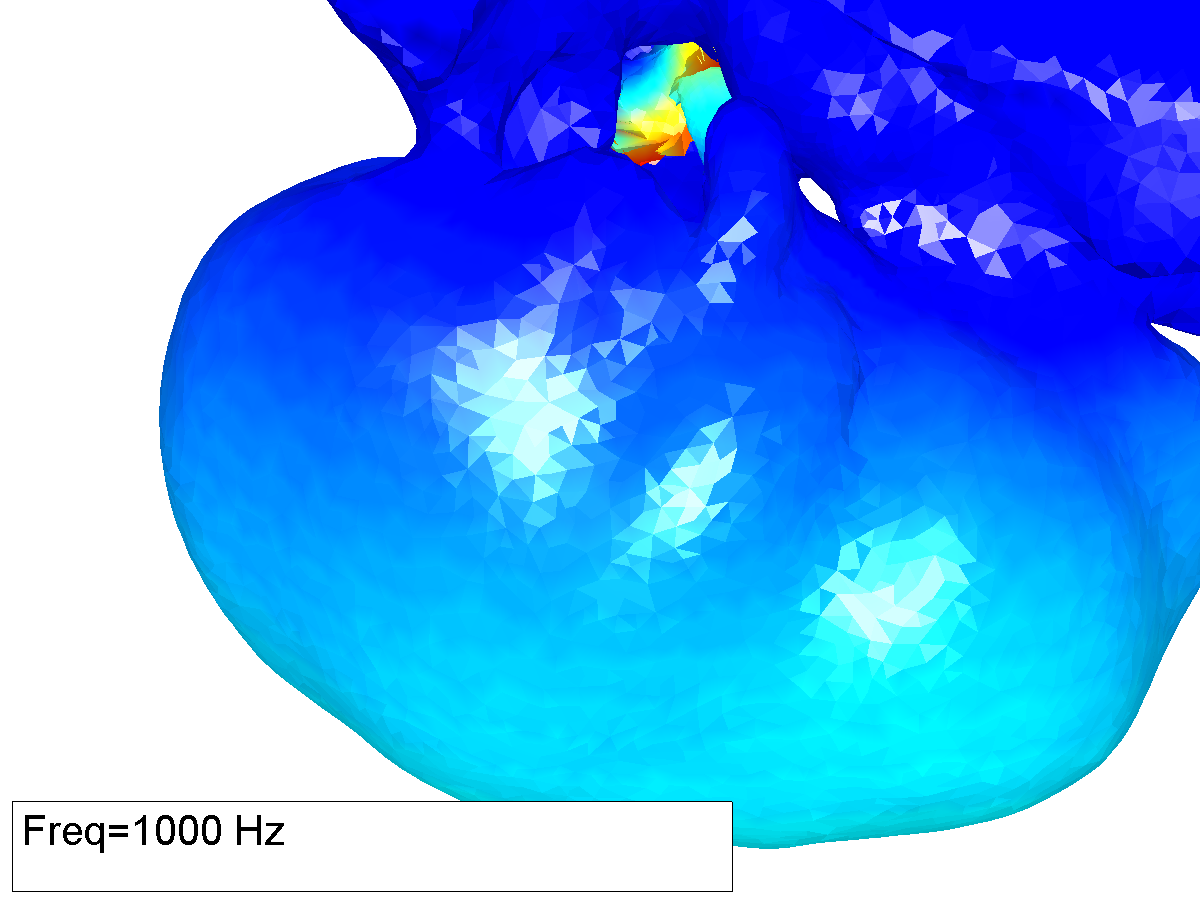


S20 Figure. Motion of the TPC for skull-vibration loading at 1 kHz. ([Animated visualization link with displacements magnified by 5000 times](http://journals.plos.org/plosone/article/asset?unique&id=info:doi/10.1371/journal.pone.0116222.s021)).


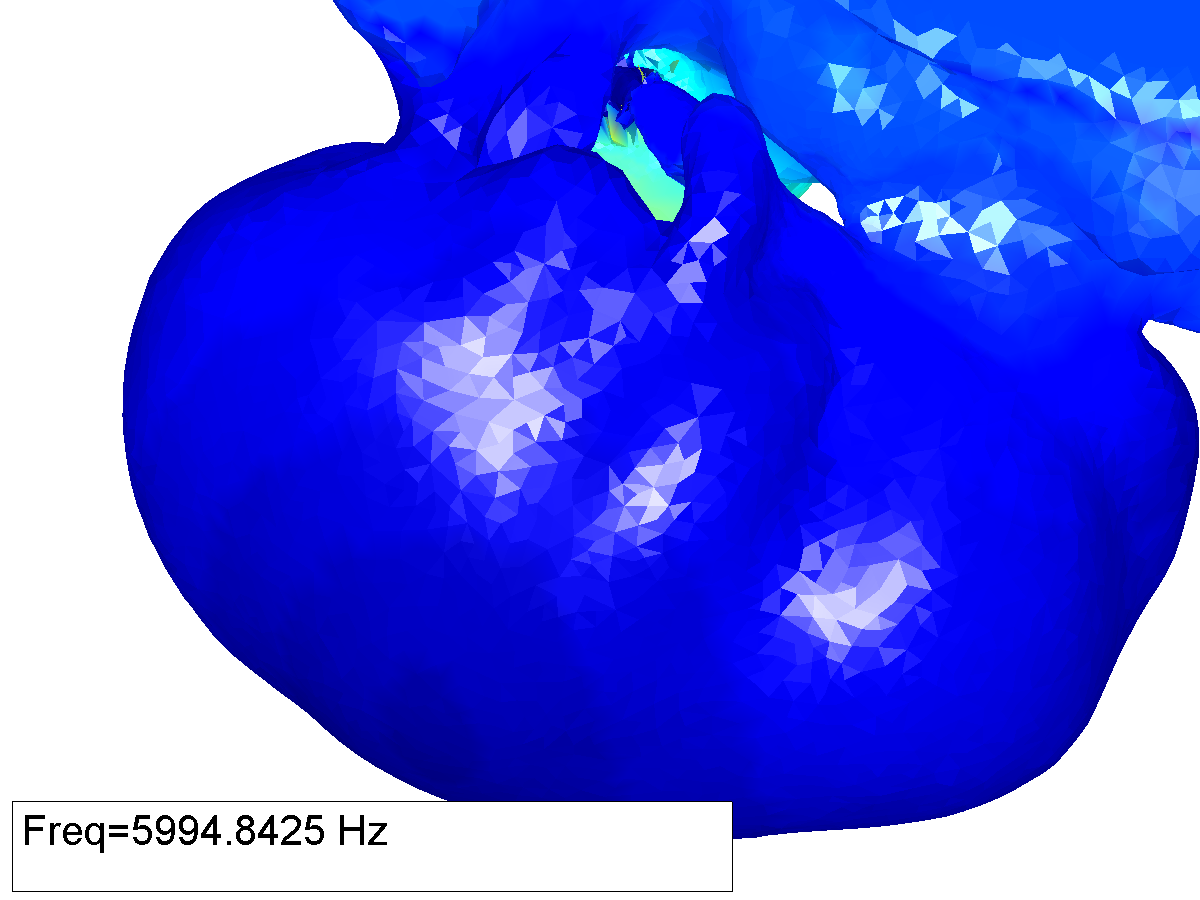


S21 Figure. Motion of the TPC for skull-vibration loading at 5.99 kHz. ([Animated visualization link with displacements magnified by 5000 times](http://journals.plos.org/plosone/article/asset?unique&id=info:doi/10.1371/journal.pone.0116222.s022)).


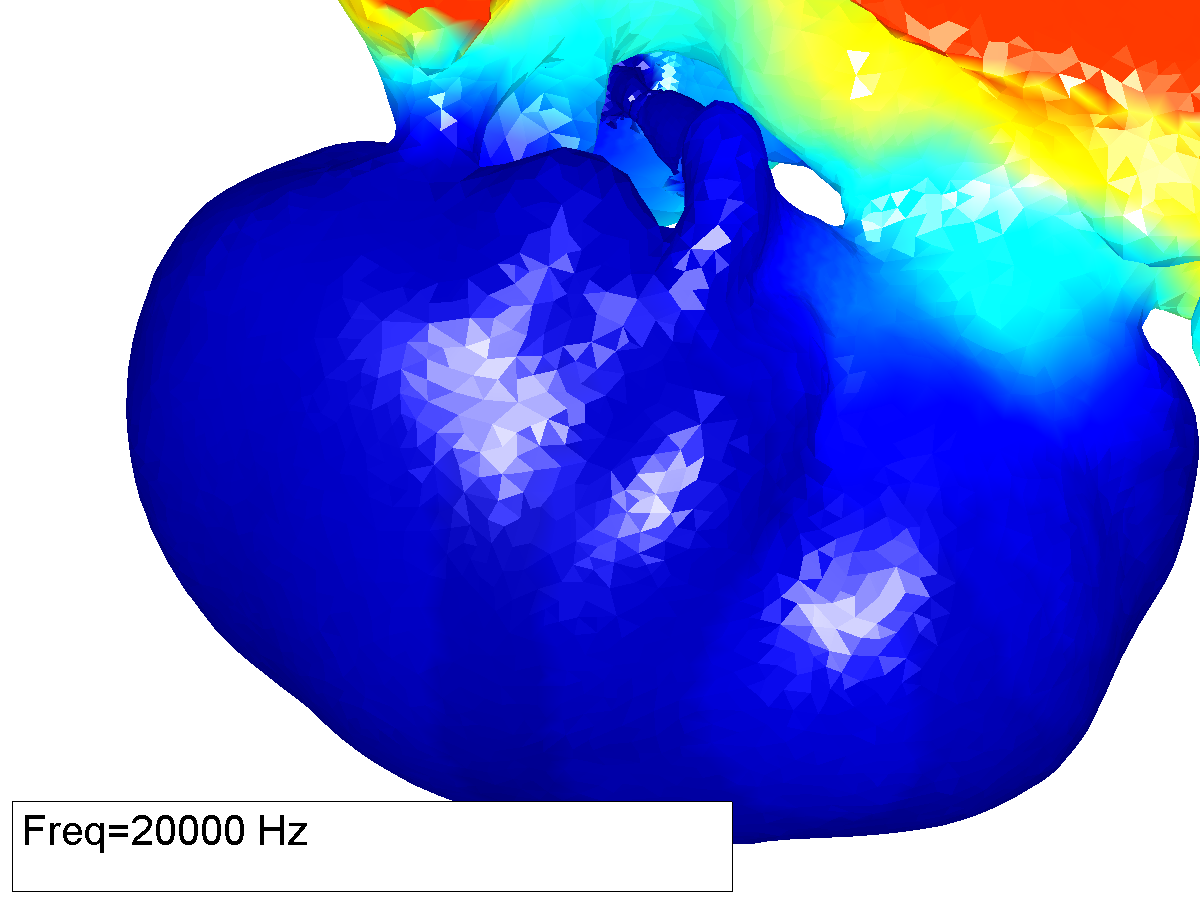


S22 Figure. Motion of the TPC for skull-vibration loading at 20 kHz. ([Animated visualization link with displacements magnified by 5000 times](http://journals.plos.org/plosone/article/asset?unique&id=info:doi/10.1371/journal.pone.0116222.s023)).

## Sensitivity studies

To assess the sensitivity of the calculated SVTF curve to the various input parameters we define the measure

$\Delta=\frac{\left\|SVTF_{\Delta}-SVTF_{R}\right\|}{\left\|SVTF_{R}\right\|}$ (S13)

Here $\Delta\geq0$ quantifies how the *SVTF* obtained for inputs changed from the defaults (Delta), $SVTF_{\Delta}$, is different from the *SVTF* obtained for the default inputs, $SVTF_{R}$. The larger the value of $\Delta$, the more significant the change of the input parameter with the respect to the *SVTF*.

The following studies were performed for the SVTF produced by pressure loading on the TPC. To indicate this, the transfer function is labeled “SVTF(P)”.

#### Effect of changes of the cochlear impedance

Changing the constant of the resistive cochlear damping by a factor of ½ (decrease) or 2 (increase) respectively has little effect: refer to Error: Reference source not found **Figure**.

S23 Figure. Change of the SVTF(P) due to a change in the cochlear impedance. Dotted line: decrease by a factor of ½, $\Delta=0.159$; dashed line: increase by a factor of 2, $\Delta=0.084$.

#### Effect of changes of the joint elastic modulus

The elastic modulus of the annular stapedial ligament and the ligaments of the joints in the ossicular chain shows a pronounced effect for frequencies above roughly 1 kHz (it should be noted however that the displayed variation of the SVTF is for rather substantial changes of the modulus: either reduced with a factor of 1/2 or increased with a factor of two with respect to the reference value). The changes can be tracked to this part of the transfer function being strongly affected by the vibration modes of the ossicles themselves (Error: Reference source not found **Figure**).

S24 Figure. Change of the SVTF(P) due to a change in the elastic modulus of the joints in the ossicular chain. Dotted line: decrease by a factor of ½, $\Delta=0.65$; dashed line: increase by a factor of 2, $\Delta=0.85$.

#### Effect of changes of the Rayleigh system damping

The amount of Rayleigh system damping depends on the two parameters, $\varsigma_{\min}$ and $\omega_{\min}$, from equation (S3). Error: Reference source not found **Figure** and Error: Reference source not found **Figure** show the changes in the SVTF(P) due to a change in the Rayleigh system damping, the modal damping ratio and the frequency of minimum damping respectively. In either case, the SVTF is affected mostly at high frequencies, ie. above 2 kHz).

S25 Figure. Change of the SVTF(P) due to a change in the Rayleigh system damping. Dotted line: decrease of $\varsigma_{\min}$ by a factor of ½, $\Delta=0.25$; dashed line: increase of $\varsigma_{\min}$ by a factor of 2, $\Delta=0.24$.

S26 Figure. Change of the SVTF(P) due to a change in the Rayleigh system damping. Dotted line: decrease of $\omega_{\min}$ by a factor of ½, $\Delta=0.21$; dashed line: increase of $\omega_{\min}$ by a factor of 2, $\Delta=0.22$.

#### Effect of changes of the damping of the tympanic bone

For the skull bone-conduction simulation the tympanic bone surface is associated with a damping term. The surface impedance was adopted at the value corresponding to seawater, ramped down to zero for zero frequency as explained above. Error: Reference source not found **Figure** shows the effect that scaling the impedance has on the transfer function for the bone-conduction forcing. Apparently, the transfer function is affected mostly at low frequencies, where increased impedance value increases the excitation at the stapes and vice versa.

S27 Figure. Change of the SVTF(U) due to a change in the surface impedance on the tympanic bone. Dotted line: decrease by a factor of ½, $\Delta=0.139$; dashed line: increase by a factor of 2, $\Delta=0.144$.

## Prediction of the audiogram

With the SVTF at hand, we can attempt to predict the audiogram. To proceed we need to calibrate the audiogram curve with the respect to the minimum audible pressure. For instance, we could assume the hearing threshold to be similar to that measured for toothed whales, the bottlenose dolphin (Johnson, 1968), or the killer whale (Szymanski et al., 1999), around 70 dB re $1\mu\text{Pa}$. Setting $SPL_{\text{th}}=70$ dB, the minimum threshold pressure across all frequencies can be estimated as

$p_{\text{inc,th,min}}=10^{(SPL_{\text{th}}/20)}p_{\text{ref}}=0.0032 [\text{Pa],}$ (S14)

and the stapes velocity at the threshold can be consequently estimated as

$SVTF_{\text{th}}=p_{\text{inc,th,min}}\max[SVTF(\omega)].$ (S15)

The threshold pressure amplitude $p_{\text{inc,th,min}}$ can be predicted as a function of frequency $\omega$ as

$p_{\text{inc,th}}(\omega)=p_{\text{inc,th,min}}\frac{\max[SVTF(\omega)]}{SVTF(\omega)}$ (S16)

#### Audiogram for the pressure loading on the TPC

For the SVTF shown in Error: Reference source not found **Figure** (A) the substitution of the minimum threshold pressure amplitude and the peak (threshold) stapes velocity results in

$SVTF_{\text{th}}(P)=p_{\text{inc,th,min}}\max[SVTF(P,\omega)]=0.0032\times1605.3\approx5.07 [\text{nm}\cdot\text{s}^{-1}].$ (S17)

This number is comparable to numbers obtained for instance for humans (Gelfand, 2004). The graph of this relationship is shown in Error: Reference source not found **Figure** (B).

(A) (B)

S28 Figure. (A) The SVTF(P). (B) The audiogram predicted from the SVTF(P).

#### Audiogram for the “skull bone conduction”

The SVTF for skull-vibration bone conduction (forcing by prescribed displacement at the periotic bone), as indicated by “(U)”, is shown in Error: Reference source not found **Figure** (A). The corresponding audiogram is constructed as in the previous section and shown in Error: Reference source not found **Figure** (B). The substitution of the minimum threshold pressure amplitude and the peak (threshold) stapes velocity results in

$SVTF_{\text{th}}(U)=p_{\text{inc,th,min}}\max[SVTF(U,\omega)]=0.0032\times4228.6\approx13.37 [\text{nm}\cdot\text{s}^{-1}].$ (S18)

The peak stapes velocity for the skull-vibration loading of the TPC is higher than that obtained above for the pressure loading. We would consequently predict this mechanism to be more effective of the two in transferring sound energy from the water to the inner ear.

#### Audiogram for the combination of the pressure and skull-vibration loading

The audiogram for the combined mechanism of floating of the TPC by both the pressure conducted by the soft tissues toward the ear bones and by the skull-vibration mechanism is constructed from the composite SVTF which is the simple sum of the individual transfer functions

$SVTF(C)=SVTF(P)+SVTF(U)$. (S19)

The audiogram curve was presented in the main text in Figure 4.

(A) (B)

S29 Figure. (A) The SVTF(U). (B) The audiogram predicted from the SVTF(U).

## Tables

S1 Table. Properties of materials used in the TPC simulations.

| Type | Young’s modulus [MPa] | Mass density [kg.m^-3^] | Poisson ratio |
| --- | --- | --- | --- |
| TPC bone | 30000 (Currey, 1979; Tubelli et al., 2012) | 2350 (Nummela et al., 1999) | 0.3 (Currey, 1979) |
| Ligament of incudomallear/incudostapedial  joint | 6.0 (Cai et al., 2010) | 1200 (Homma et al., 2009) | 0.47 (Zhang et al., 2011) |
| Annular stapedial ligament | 0.1 (Gan et al., 2011) | 1200 (Homma et al., 2009) | 0.47 (Zhang et al., 2011) |

S2 Table. Properties of materials used in the VATk simulations.

| Type | Young’s modulus [MPa] | Mass density [kg.m^-3^] | Poisson ratio |
| --- | --- | --- | --- |
| TPC (ear) bone | 30000 (Currey, 1979; Tubelli et al., 2012) | 2350 (Nummela et al., 1999) | 0.3 (Currey, 1979) |
| Skull bone | 19000 | 2300 | 0.3 |
| Soft tissue | 0.1 | 993 | 0.49999254 |

**References**

Aibara, R., Welsh, J., Puria, S., Goode, R. 2001. Human middle-ear sound transfer function and cochlear input impedance. Hearing Research 152, 100-109.

Cai, H., Jackson, R., Steele, C., Puria, S. 2010. A Biological Gear in the Human Middle Ear, Comsol conference, Boston.

Castellazzi, G., Krysl, P., Rojas, L., Cranford, T.W. 2012. Assessment of the Effect of Natural and Anthropogenic Aquatic Noise on Vaquita (*Phocoena sinus*) Through a Numerical Simulation. In: Popper, A.N., Hawkins, A.D., (Eds.), Effects of Noise on Aquatic Life, Vol. 730. Springer Science+Business Media, LLC, New York. pp. 307-310.

Cranford, T.W. 1999. The sperm whale's nose: Sexual selection on a grand scale? Marine Mammal Science: Notes 15, 1133-1157.

Cranford, T.W., Krysl, P. 2012. Acoustic function in the peripheral auditory system of Cuvier’s Beaked Whale (*Ziphius cavirostris*). In: Popper, A.N., Hawkins, A.D., (Eds.), Effects of Noise on Aquatic Life, Vol. 730. Springer Science+Business Media, LLC, New York. pp. 69-72.

Cranford, T.W., Krysl, P., Hildebrand, J.A. 2008a. Acoustic pathways revealed: Simulated sound transmission and reception in Cuvier’s beaked whale (*Ziphius cavirostris*). Bioinspiration & Biomimetics 3, e016001.

Cranford, T.W., McKenna, M.F., Soldevilla, M.S., Wiggins, S.M., Shadwick, R.E., Goldbogen, J., Krysl, P., St. Leger, J.A., Hildebrand, J.A. 2008b. Anatomic geometry of sound transmission and reception in Cuvier's beaked whale (*Ziphius cavirostris*). Anat. Rec. 291, 353-378.

Currey, J.D. 1979. Mechanical properties of bone tissues with greatly differing functions. Journal of Biomechanics 12, 313-319.

Gan, R., Yang, F., Zhang, X., Nakmali, D. 2011. Mechanical properties of stapedial annular ligament. Medical Engineering & Physics 33, 330-339.

Gelfand, S. 2004. Hearing: An Introduction to Psychological and Physiological Acoustics, Fourth Edition Taylor & Francis.

Homma, K., Kim, N., Puria, S. 2011. Towards Creation of a Human-head Auditory Model for Simulating Bone-Conduction Pathways. AIP Conference Proceedings 1403, 552-553.

Homma, K., Du, Y., Shimizu, Y., Puria, S. 2009. Ossicular resonance modes of the human middle ear for bone and air conduction. Journal of the Acoustical Society of Americ 125, 968-979.

Johnson, C.S. 1968. Masked tonal thresholds in the bottlenosed porpoise. The Journal of the Acoustical Society of America 44, 965-967.

Krysl, P. 2011. Thermal and Stress Analysis with the Finite Element Method San Diego: Pressure Cooker Press, San Diego.

Krysl, P., Zhu, B. 2008a. Locking-free continuum displacement finite elements with nodal integration. International Journal for Numerical Methods In Engineering 76, 1020-1043.

Krysl, P., Kagey, H. 2012a. Reformulation of nodally integrated continuum elements to attain insensitivity to distortion. International Journal for Numerical Methods In Engineering 90, 805-818.

Krysl, P., Cranford, T.W., Hildebrand, J.A. 2008b. Lagrangian finite element treatment of transient vibration/acoustics of biosolids immersed in fluids. Int. J. Numer. Meth. Engng. 74, 754-775.

Krysl, P., Trijoulet, V., Cranford, T.W. 2012b. Validation of a vibroacoustic finite-element model using bottlenose dolphin experiments. In: Popper, A.N., Hawkins, A.D., (Eds.), Effects of Noise on Aquatic Life, Vol. 730. Springer Science+Business Media, LLC, New York. pp. 65-68.

Nummela, S., Wägar, T., Hemilä, S., Reuter, T. 1999. Scaling of the cetacean middle ear. Hearing Research 133, 71-81.

Oberrecht, S., Krysl, P., Cranford, T.W. 2014. Sound transmission validation and sensitivity studies in numerical models. In: Popper, A.N., Hawkins, A.D., (Eds.), Effects of Noise on Aquatic Life II. Springer Science+Business Media, LLC, New York.

Puria, S., Rosowski, J. 2012. Bekesy's contributions to our present understanding of sound conduction to the inner ear. Hearing Research 293, 21-30.

Szymanski, M.D., Bain, D.E., Kiehl, K., Pennington, S., Wong, S., Henry, K.R. 1999. Killer whale (*Orcinus orca*) hearing: Auditory brainstem response and behavioral audiograms. Journal of the Acoustical Society of Americ 106, 1134-1141.

Tubelli, A.A., Zosuls, A., Ketten, D.R., Yamato, M., Mountain, D.C. 2012. A prediction of the minke whale (*Balaenoptera acutorostrata*) middle-ear transfer function. The Journal of the Acoustical Society of America 132, 3263-3272.

Volandri, G., Di Puccio, F., Forte, P., Manetti, S. 2012. Model-oriented review and multi-body simulation of the ossicular chain of the human middle ear. Medical Engineering & Physics 34, 1339-1355.

Yamato, M., Ketten, D.R., Arruda, J., Cramer, S., Moore, K. 2012. The auditory anatomy of the minke whale (*Balaenoptera acutorostrata*): a potential fatty sound reception pathway in a baleen whale. Anat. Rec. 295, 991-998.

Zhang, X., Gan, R. 2011. Experimental measurement and modeling analysis on mechanical properties of incudostapedial joint. Biomechanics and Modeling in Mechanobiology 10, 713-726.
