# Supplementary figures and images for: Fin Whale Sound Reception Mechanisms: Skull Vibration Enables Low-Frequency Hearing
Source: PLoS One. 2015 Jan 29;10(1):e0116222. doi: 10.1371/journal.pone.0116222 (PMC4310601; doi:10.1371/journal.pone.0116222)

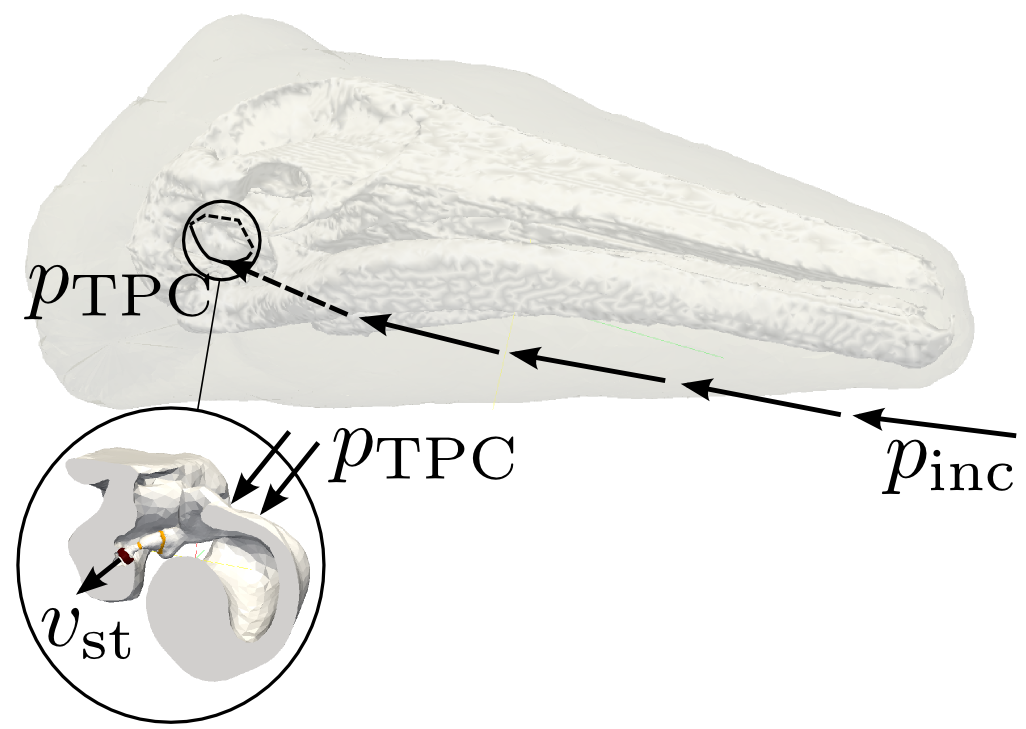

Supplement: S1 Fig — (PNG) [file pone.0116222.s002.png]

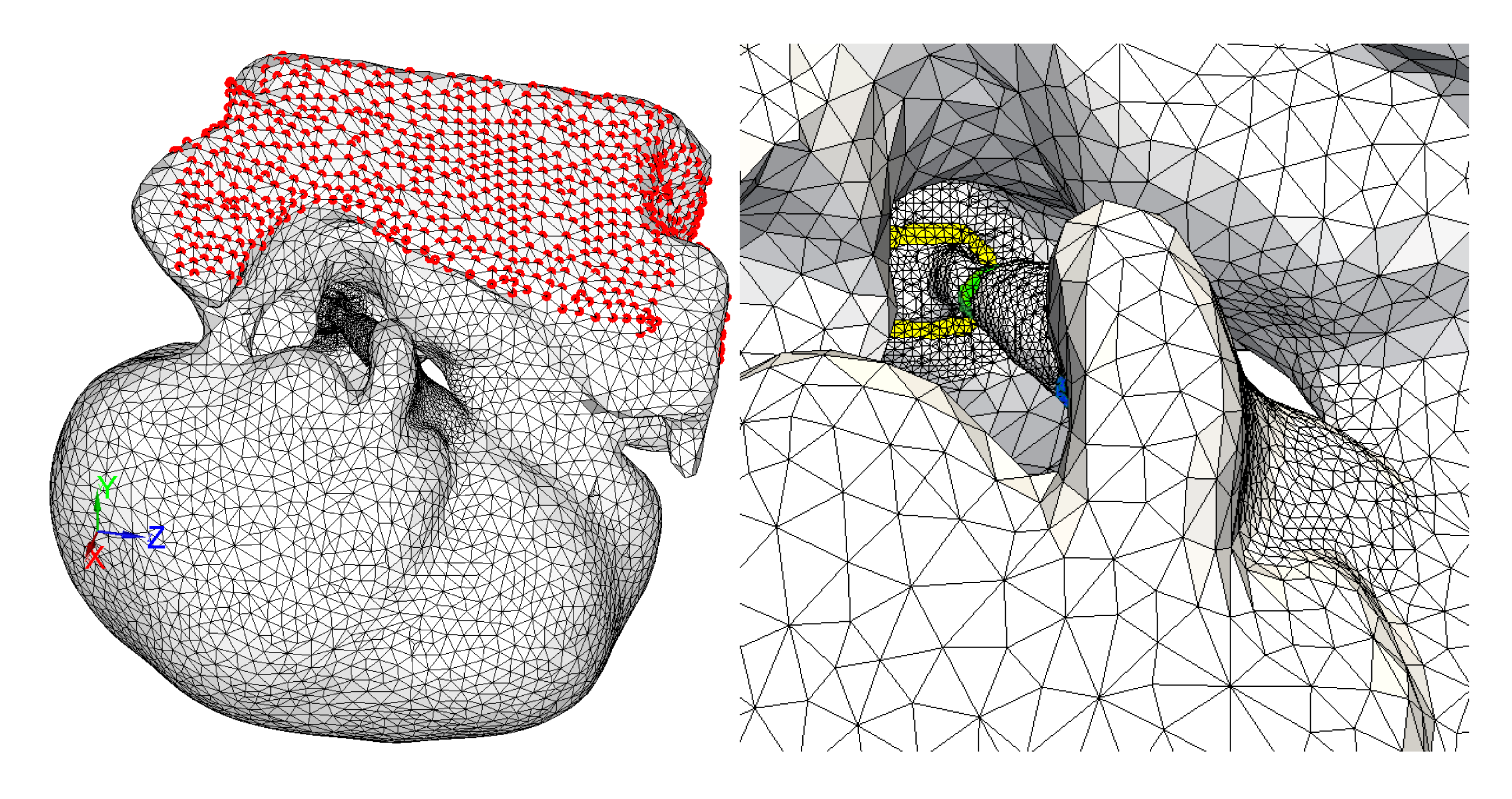

Supplement: S2 Fig — (A) Finite element mesh 4 (approximately 41,000 nodes, 230,000 elements). The periotic bone is trimmed off, and the red markers at the top-right of the mesh indicate nodes with prescribed displacements (as dictated by the motion or the lack of the motion of the squamosal bone of the skull). (B) Close-up of the ossicular chain and the sigmoidal process in the foreground. The joints between the ossicles are shown in color: the annular ligament between the stapes and the oval window is yellow; the incudostapedial ligament is green, and a small portion of the malleoincudal ligament is blue (most of this ligament and the malleus are obscured by the sigmoidal process). (TIF) [file pone.0116222.s003.tif]

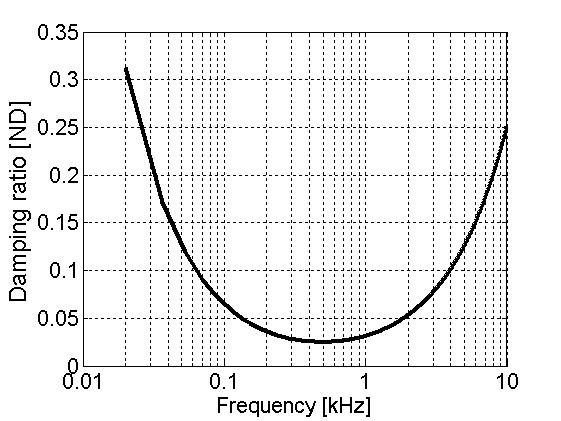

Supplement: S3 Fig — (PNG) [file pone.0116222.s004.png]

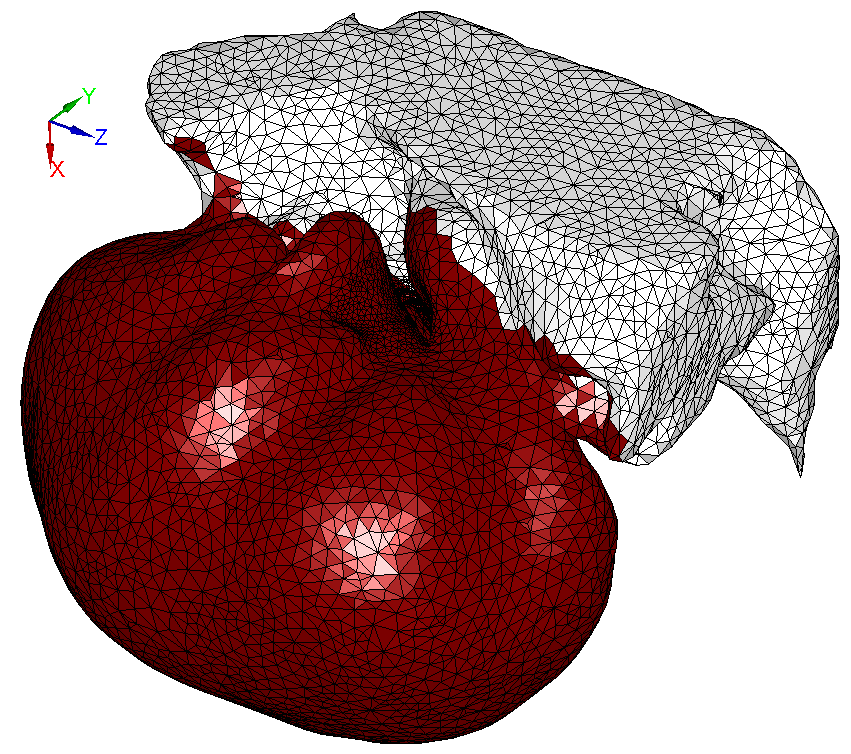

Supplement: S4 Fig — Mesh 4 as in S2 Fig. (PNG) [file pone.0116222.s005.png]

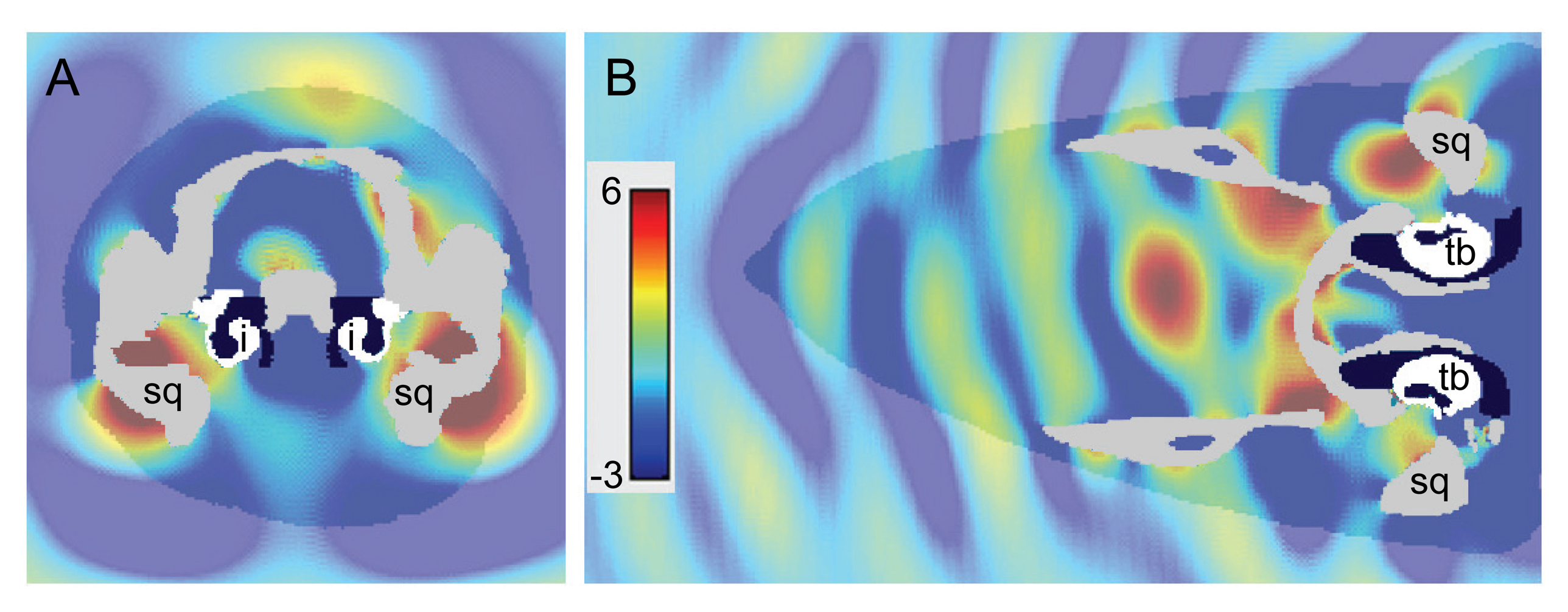

Supplement: S5 Fig — Sound Pressure Levels (SPL) between -3 dB (pressure diminished with the respect to incident) and +6 dB (pressure amplified with respect to incident) are shown. The pressure is not displayed in the volume of the bones (gray regions) or in the volume of the air spaces or sinuses (black regions). (A) is a transverse section through the TPC with labels indicating the involucra (i) of the tympanic bulla and the expanded portion of the squamosal (sq); (B) is a coronal (horizontal) section through the TPC, at the level of the tympanic bullae (tb). Note the amplified pressure amplitude near the dorsal surface of the tympanic bullae (tb) from a reflection off of the squamosal bones (sq). (PNG) [file pone.0116222.s006.png]

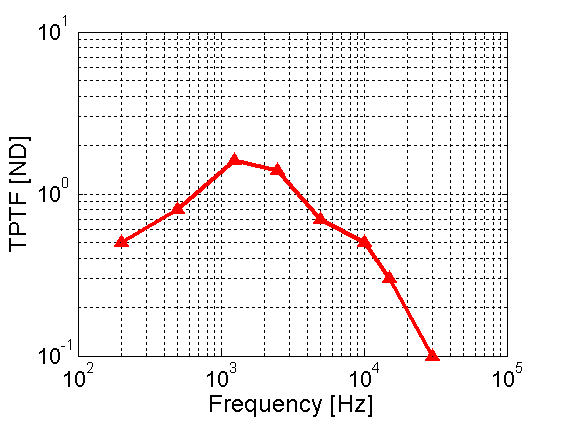

Supplement: S6 Fig — Note that close to 1–2 kHz the incident pressure is magnified to arrive at the surface of the TPC almost doubled in amplitude. (PNG) [file pone.0116222.s007.png]

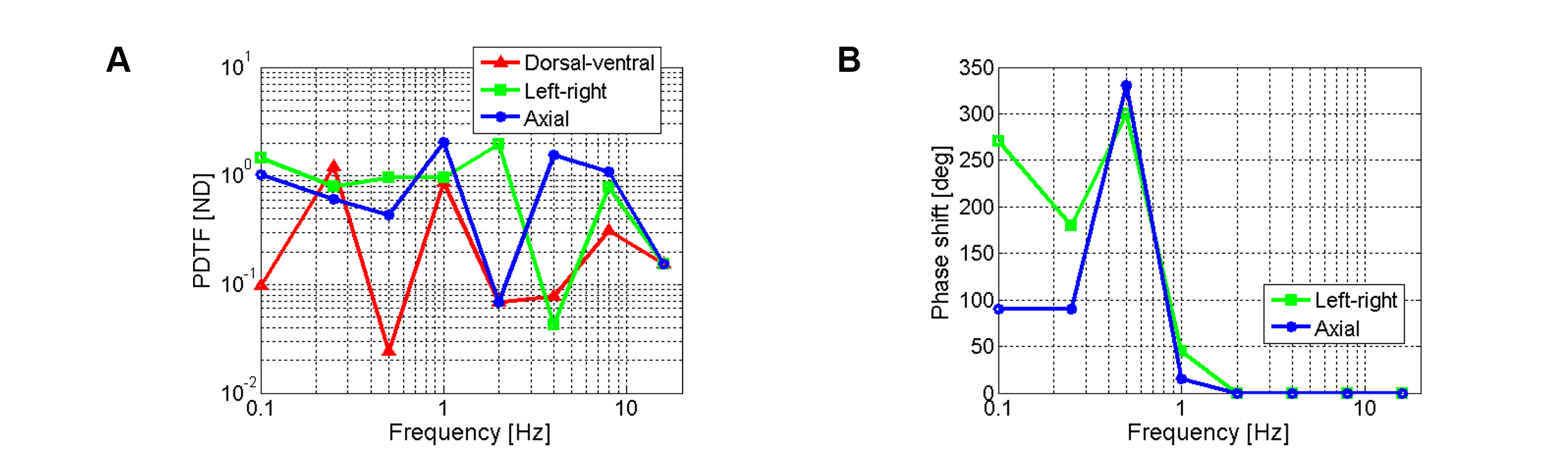

Supplement: S7 Fig — (A) Amplitudes of the displacements, and (B) phase shift with respect to the dorsal-ventral displacement. (TIF) [file pone.0116222.s008.tif]

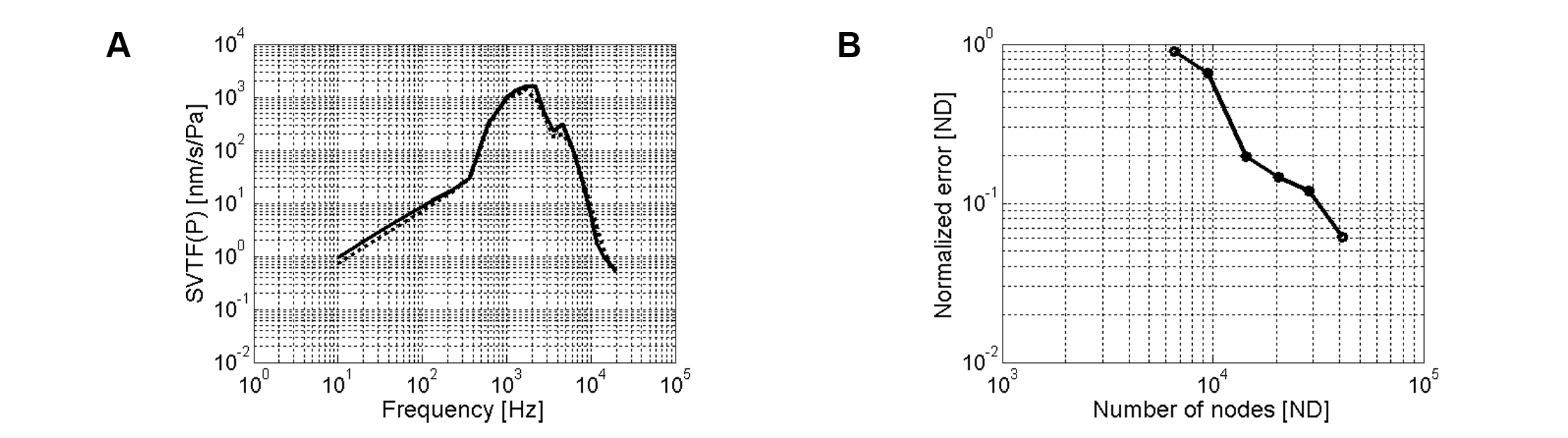

Supplement: S8 Fig — (A) SVTF(P) for two meshes: mesh 3, with 62,000 nodes, in solid line, and mesh 6, with 20,600 nodes, in dashed line. (B) Approximate error vs. the number of nodes in the model, where the smallest error is E a,4 = 0.061. The errors are for meshes 4,…,9 (right to left). (TIF) [file pone.0116222.s009.tif]

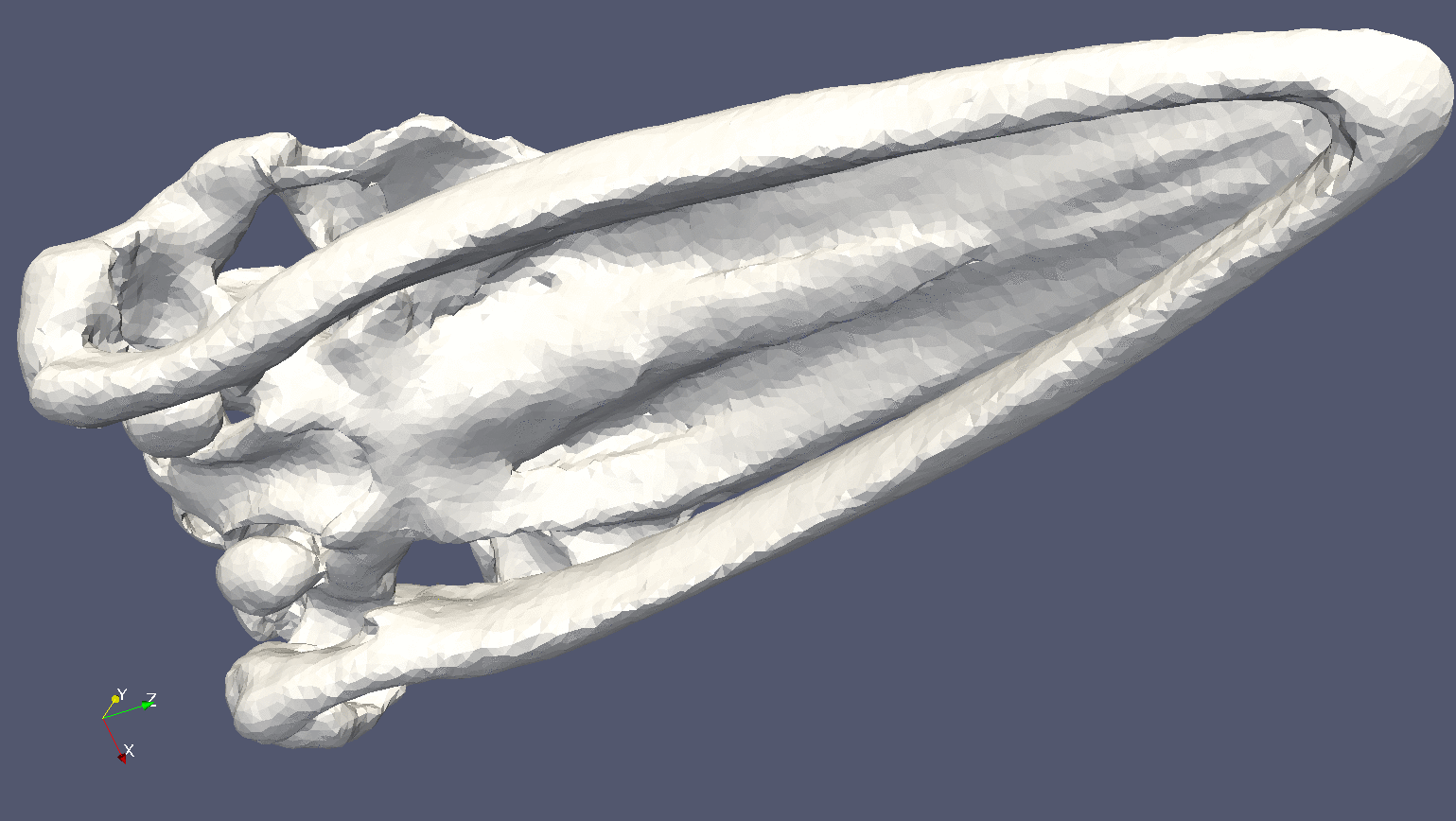

Supplement: S9 Fig — Amplitude magnified 20,000 times. (Animated visualization link with displacements magnified by 20,000 times). (GIF) [file pone.0116222.s010.gif]

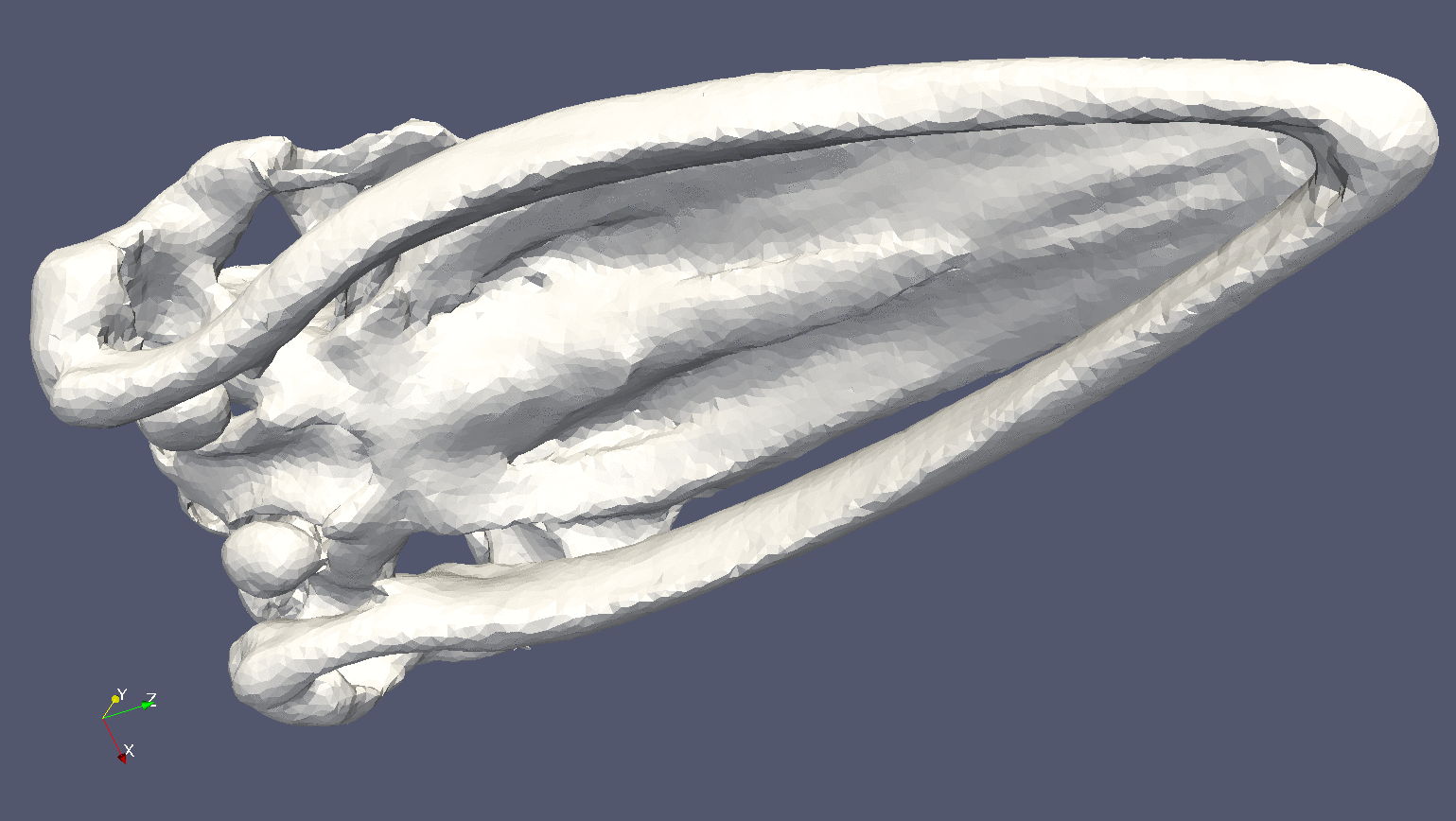

Supplement: S10 Fig — Amplitude magnified 20,000 times. (Animated visualization link with displacements magnified by 20,000 times). (GIF) [file pone.0116222.s011.gif]

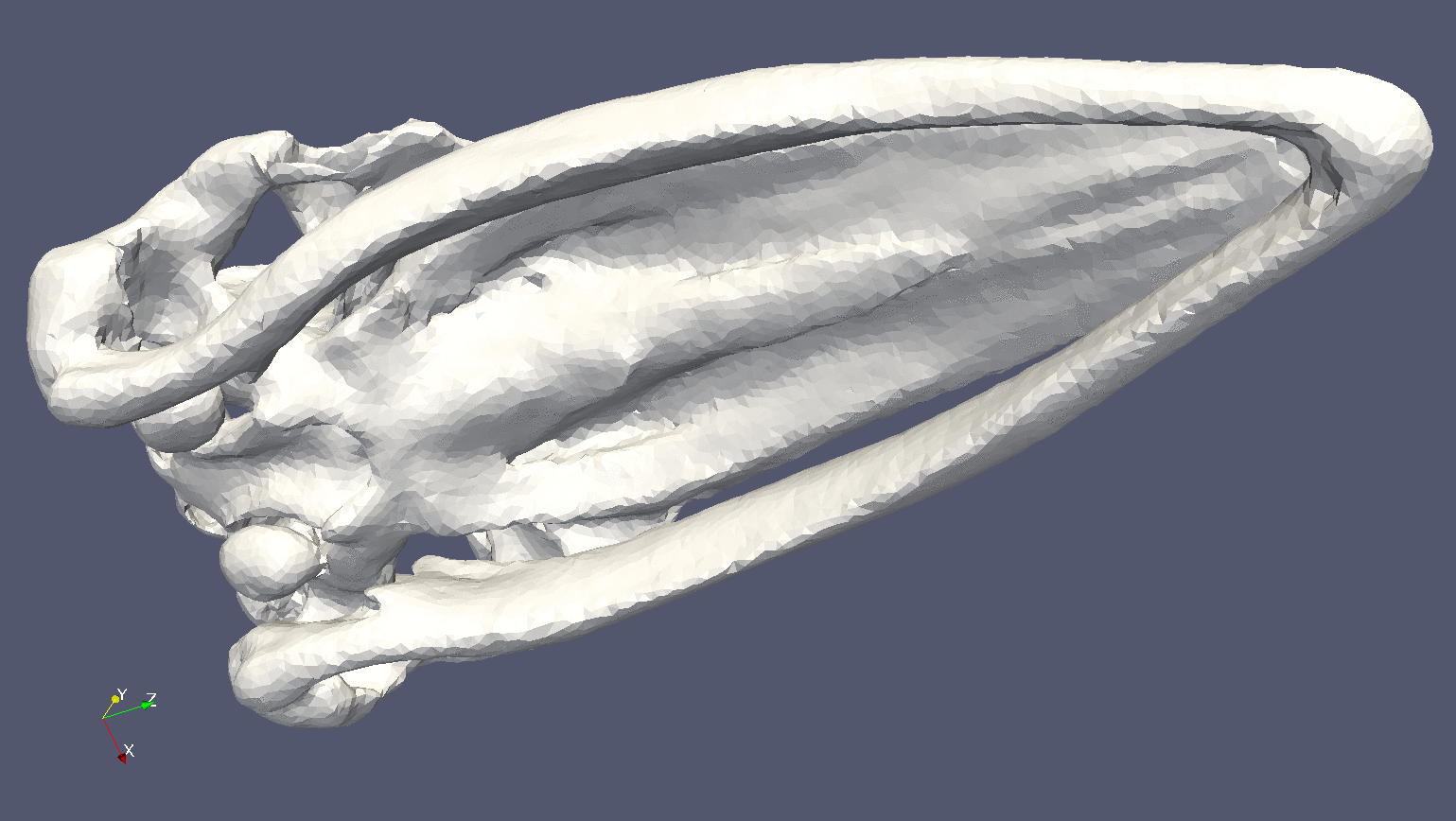

Supplement: S11 Fig — Amplitude magnified 20,000 times. (Animated visualization link with displacements magnified by 20,000 times). (GIF) [file pone.0116222.s012.gif]

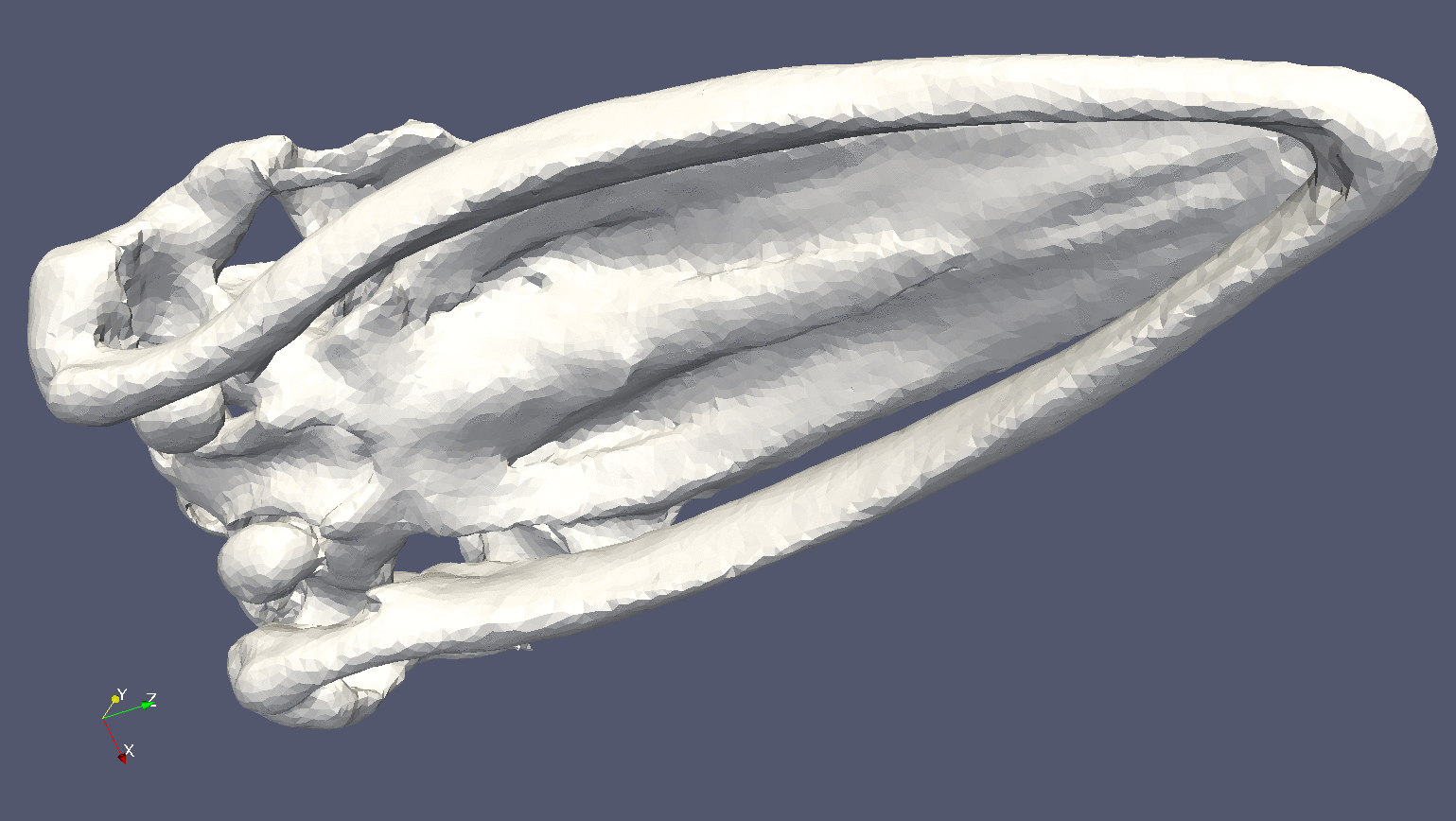

Supplement: S12 Fig — Amplitude magnified 20,000 times. (Animated visualization link with displacements magnified by 20,000 times). (GIF) [file pone.0116222.s013.gif]

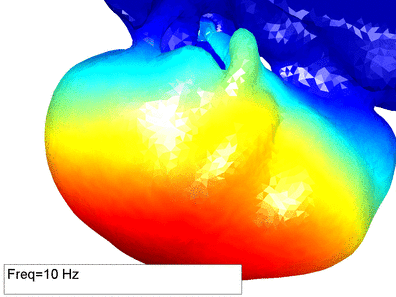

Supplement: S13 Fig — (Animated visualization link with displacements magnified by 5,000 times). (GIF) [file pone.0116222.s014.gif]

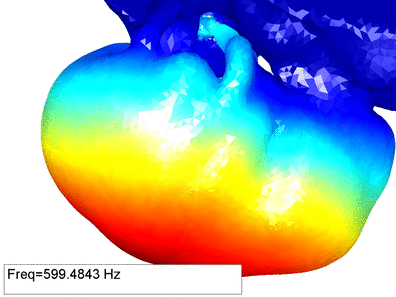

Supplement: S14 Fig — (Animated visualization link with displacements magnified by 5,000 times). (GIF) [file pone.0116222.s015.gif]

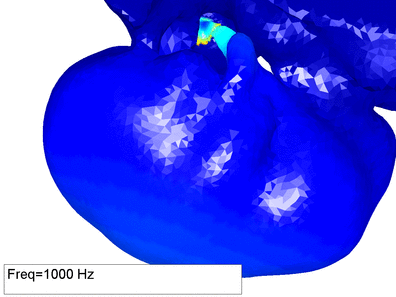

Supplement: S15 Fig — (Animated visualization link with displacements magnified by 5,000 times). (GIF) [file pone.0116222.s016.gif]

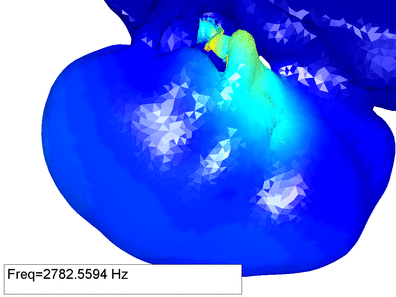

Supplement: S16 Fig — (Animated visualization link with displacements magnified by 5,000 times). (GIF) [file pone.0116222.s017.gif]

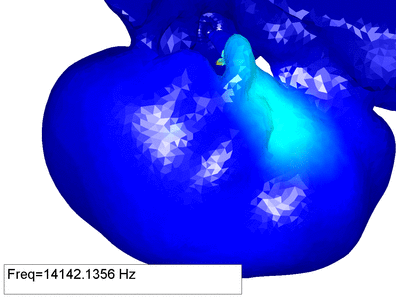

Supplement: S17 Fig — (Animated visualization link with displacements magnified by 5,000 times). (GIF) [file pone.0116222.s018.gif]

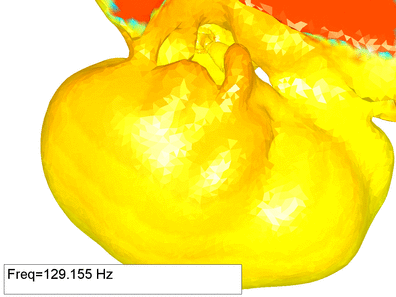

Supplement: S18 Fig — (Animated visualization link with displacements magnified by 5,000 times). (GIF) [file pone.0116222.s019.gif]

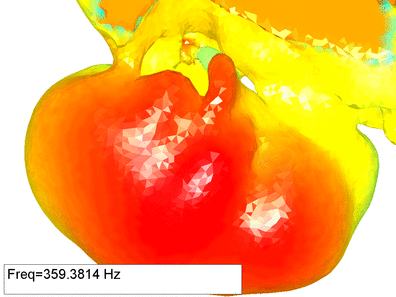

Supplement: S19 Fig — (Animated visualization link with displacements magnified by 5,000 times). (GIF) [file pone.0116222.s020.gif]

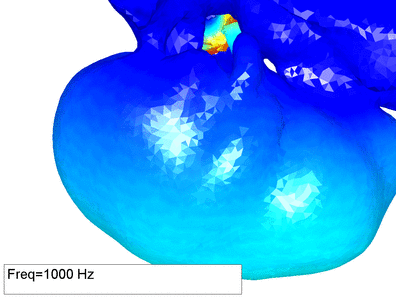

Supplement: S20 Fig — (GIF) [file pone.0116222.s021.gif]

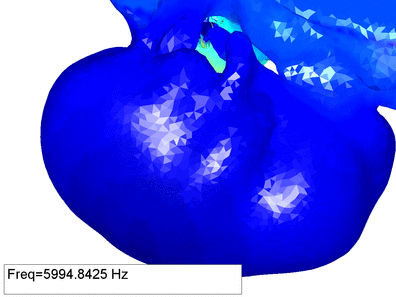

Supplement: S21 Fig — (GIF) [file pone.0116222.s022.gif]

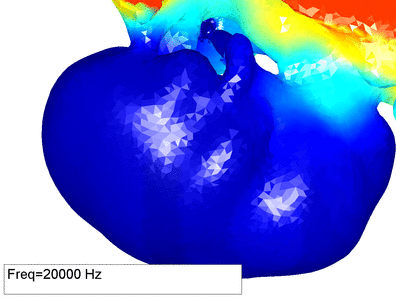

Supplement: S22 Fig — (GIF) [file pone.0116222.s023.gif]

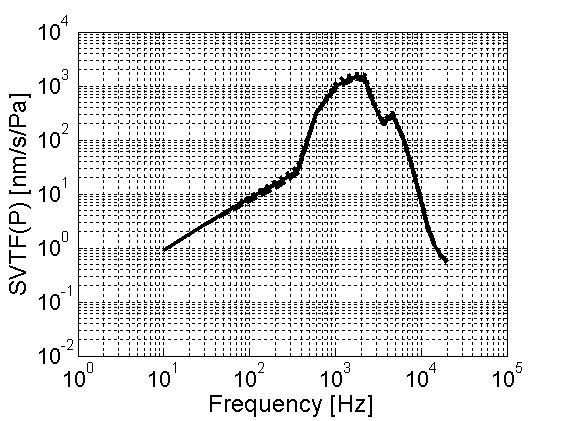

Supplement: S23 Fig — Dotted line: decrease by a factor of ½, Δ = 0.159; dashed line: increase by a factor of 2, Δ = 0.084. (PNG) [file pone.0116222.s024.png]

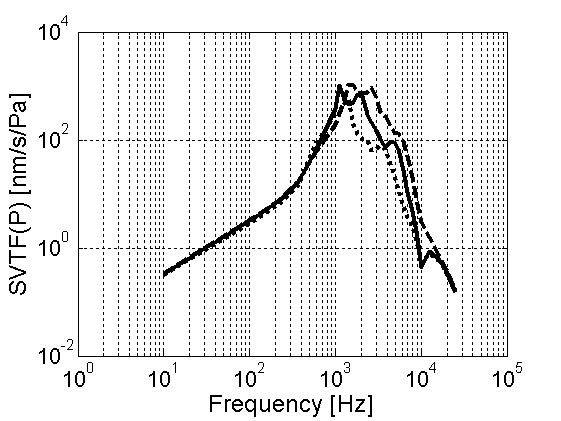

Supplement: S24 Fig — Dotted line: decrease by a factor of ½, Δ = 0.65; dashed line: increase by a factor of 2, Δ = 0.85. (PNG) [file pone.0116222.s025.png]

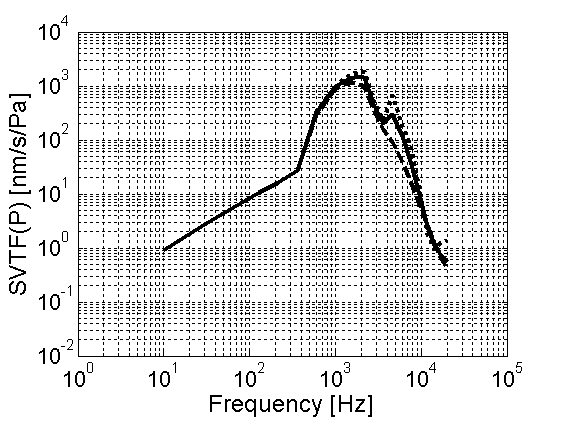

Supplement: S25 Fig — Dotted line: decrease of ςmin by a factor of ½, Δ = 0.25; dashed line: increase of ςmin by a factor of 2, Δ = 0.24. (PNG) [file pone.0116222.s026.png]

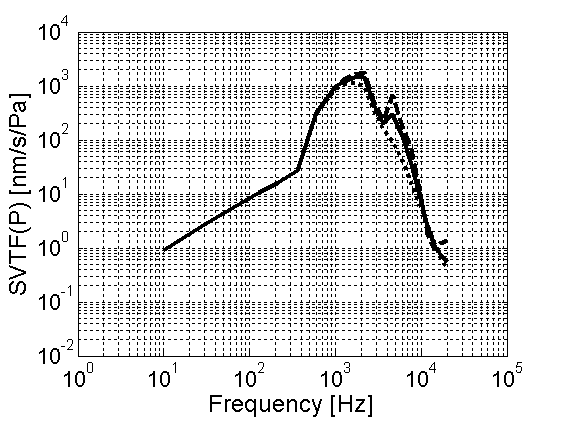

Supplement: S26 Fig — Dotted line: decrease of ω min by a factor of ½, Δ = 0.21; dashed line: increase of ω min by a factor of 2, Δ = 0.22. (PNG) [file pone.0116222.s027.png]

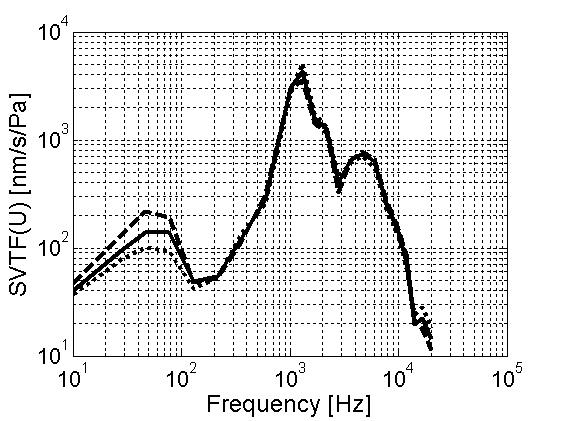

Supplement: S27 Fig — Dotted line: decrease by a factor of ½, Δ = 0.139; dashed line: increase by a factor of 2, Δ = 0.144. (PNG) [file pone.0116222.s028.png]

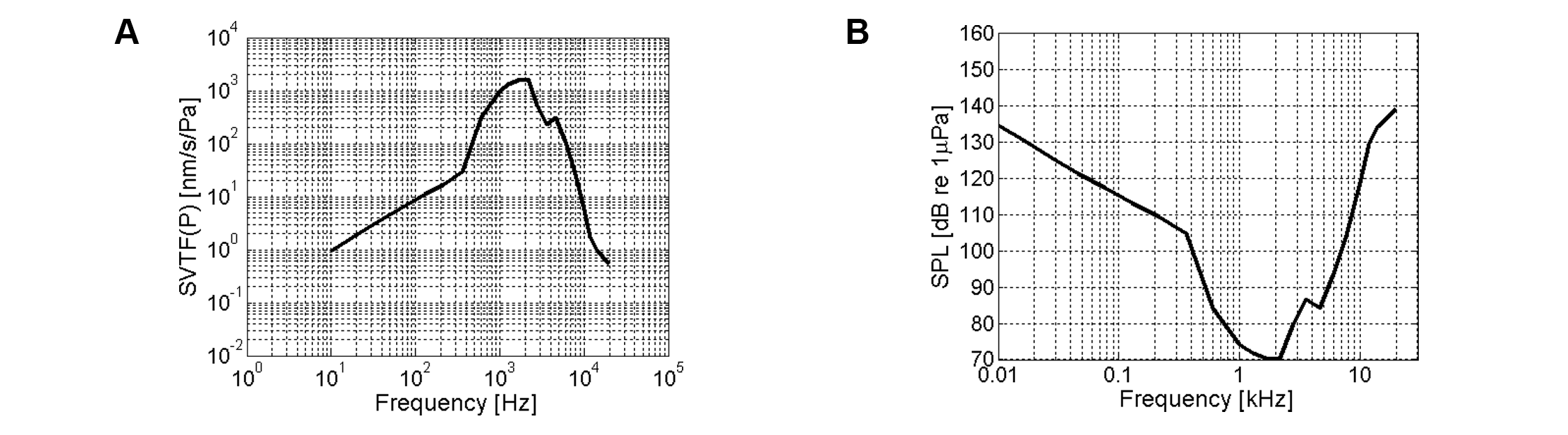

Supplement: S28 Fig — (TIF) [file pone.0116222.s029.tif]

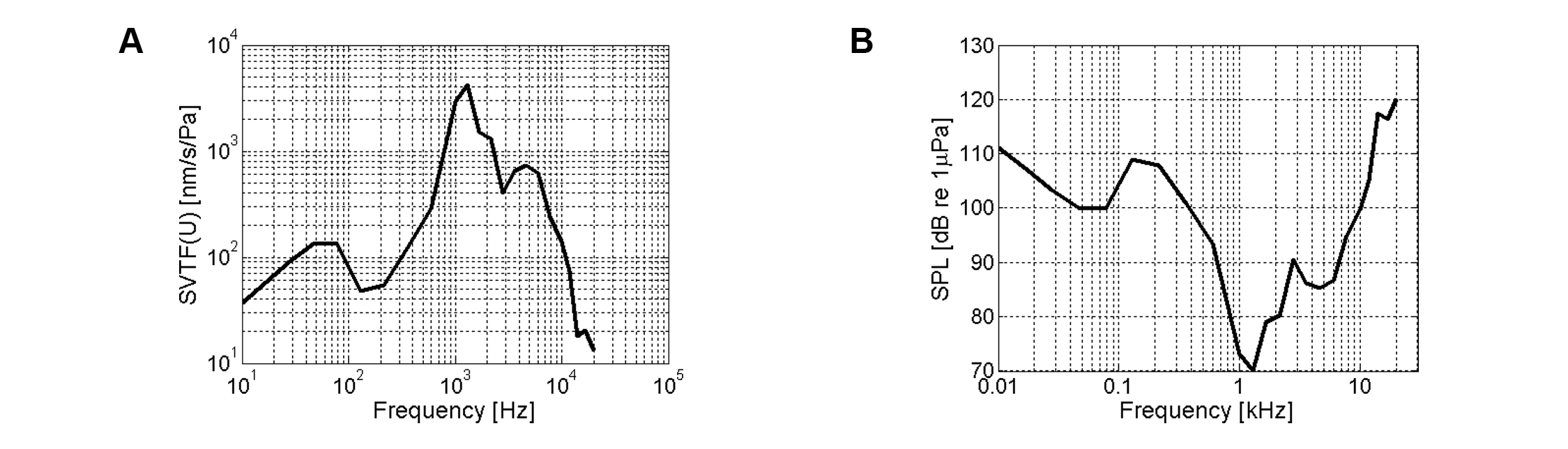

Supplement: S29 Fig — (TIF) [file pone.0116222.s030.tif]
