# Supplementary material for: Fin Whale Sound Reception Mechanisms: Skull Vibration Enables Low-Frequency Hearing
Source: PLoS One. 2015 Jan 29;10(1):e0116222. doi: 10.1371/journal.pone.0116222 (PMC4310601; doi:10.1371/journal.pone.0116222)
Supplement: S1 Table — (DOCX) [file pone.0116222.s031.docx]

S1 Table. Properties of materials used in the TPC simulations.

| Type | Young’s modulus [MPa] | Mass density [kg.m^-3^] | Poisson ratio |
| --- | --- | --- | --- |
| TPC bone | 30000 (Currey, 1979; Tubelli et al., 2012) | 2350 (Nummela et al., 1999) | 0.3 (Currey, 1979) |
| Ligament of incudomallear/incudostapedial  joint | 6.0 (Cai et al., 2010) | 1200 (Homma et al., 2009) | 0.47 (Zhang et al., 2011) |
| Annular stapedial ligament | 0.1 (Gan et al., 2011) | 1200 (Homma et al., 2009) | 0.47 (Zhang et al., 2011) |
