# Supplementary material for: Fin Whale Sound Reception Mechanisms: Skull Vibration Enables Low-Frequency Hearing
Source: PLoS One. 2015 Jan 29;10(1):e0116222. doi: 10.1371/journal.pone.0116222 (PMC4310601; doi:10.1371/journal.pone.0116222)
Supplement: S2 Table — (DOCX) [file pone.0116222.s032.docx]

S2 Table. Properties of materials used in the VATk simulations.

| Type | Young’s modulus [MPa] | Mass density [kg.m^-3^] | Poisson ratio |
| --- | --- | --- | --- |
| TPC (ear) bone | 30000 (Currey, 1979; Tubelli et al., 2012) | 2350 (Nummela et al., 1999) | 0.3 (Currey, 1979) |
| Skull bone | 19000 | 2300 | 0.3 |
| Soft tissue | 0.1 | 993 | 0.49999254 |
